# Supplementary material for: C11orf54 promotes DNA repair via blocking CMA-mediated degradation of HIF1A
Source: Commun Biol. 2023 Jun 5;6:606. doi: 10.1038/s42003-023-04957-1 (PMC10241914; doi:10.1038/s42003-023-04957-1)
Supplement: Supplementary file 2 — Supplementary Information [file 42003_2023_4957_MOESM2_ESM.pdf]

Supplementary material for C11orf54 promotes DNA repair via blocking CMA-mediated degradation of HIF1A

Junyang Tan[1,2†], Wenjun Wang[1,2†], Xinjie Liu[1,2], Jinhong Xu[1,2], Yaping Che[1,2], Yanyan Liu[1,2], Jiaqiao Hu[1,2], Liubing Hu[1,2], Jianshuang Li[1,2*] and Qinghua Zhou[1,2*]

1, The Sixth Affiliated Hospital of Jinan University, Jinan University, Dongguan, Guangdong, 523067, China

2, The Biomedical Translational Research Institute, Health Science Center (School of Medicine), Jinan University, Guangzhou, Guangdong, 510632, China

[†] These authors contributed equally.

[*] These authors jointly supervised this work.

Jianshuang Li: lijianshuan1314@jnu.edu.cn

Qinghua Zhou: gene@email.jnu.edu.cn

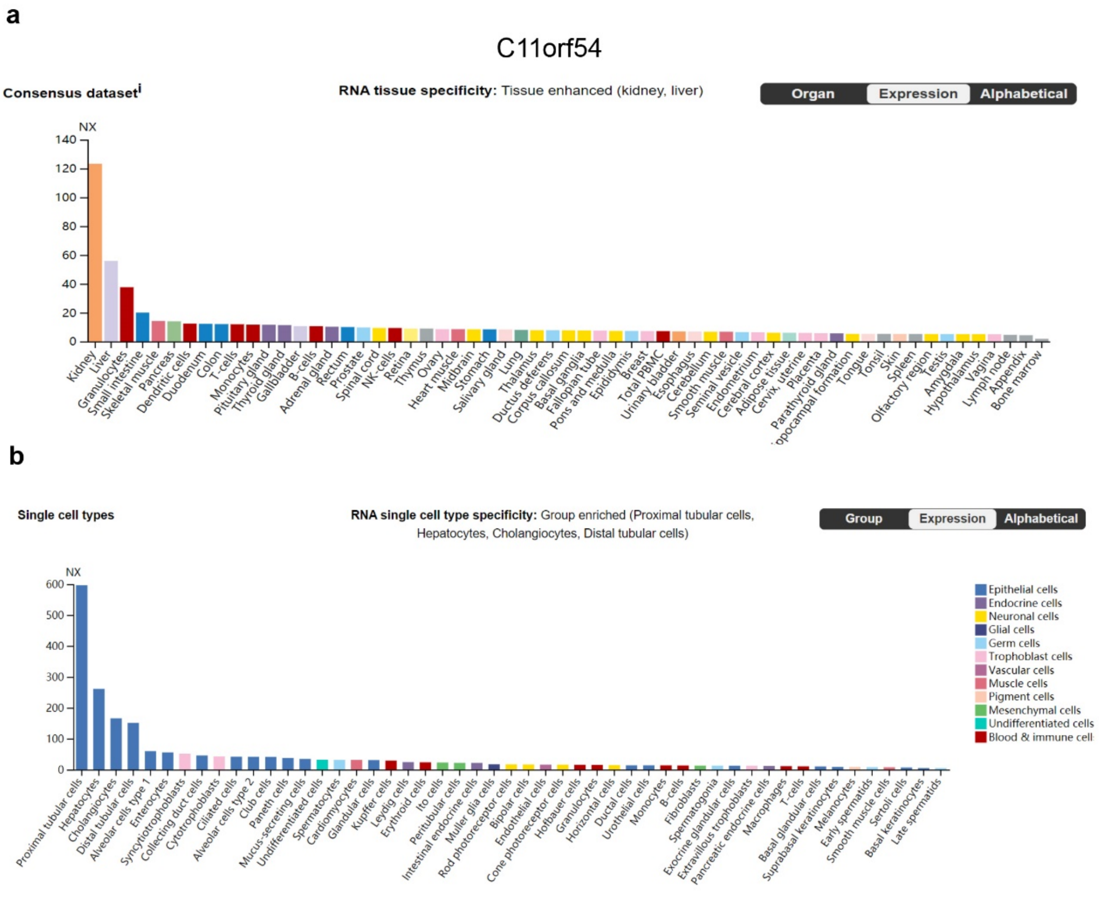

Supplementary Figure 1. Human Protein Atlas (HPA) database (https://www.proteinatlas.org/) shows that C11orf54 is enriched in kidney and liver tissue

a The expression of C11orf54 in consensus dataset consists of normalized expression levels created by combining the HPA and GTEx transcriptomics datasets.

b The expression of C11orf54 in single-cell RNA (nTPM) database.

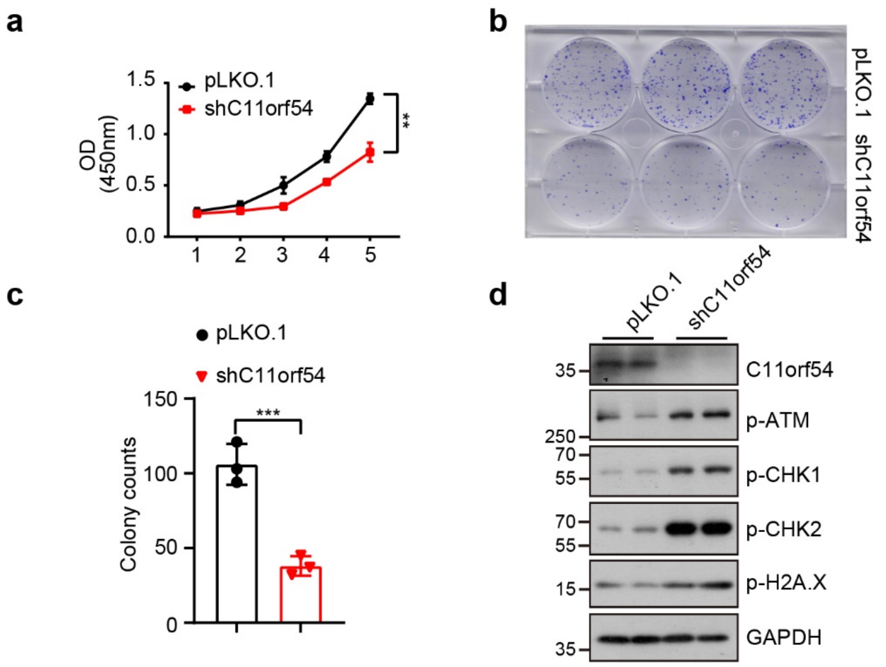

Supplementary Figure 2. C11orf54 knockdown suppresses cell proliferation and promotes DNA damage in 293T cell line

a CCK8 assay shows the cell survival of C11orf54 knockdown 293T cells and control cells.

b, c Colony formation (B) and quantitative results (C) show the cell growth of C11orf54 knockdown 293T cells and control cells (n = 3 biological replicates).

d Western blot of the indicated proteins in control and C11orf54 knockdown 293T cells (n = 3 biological replicates).

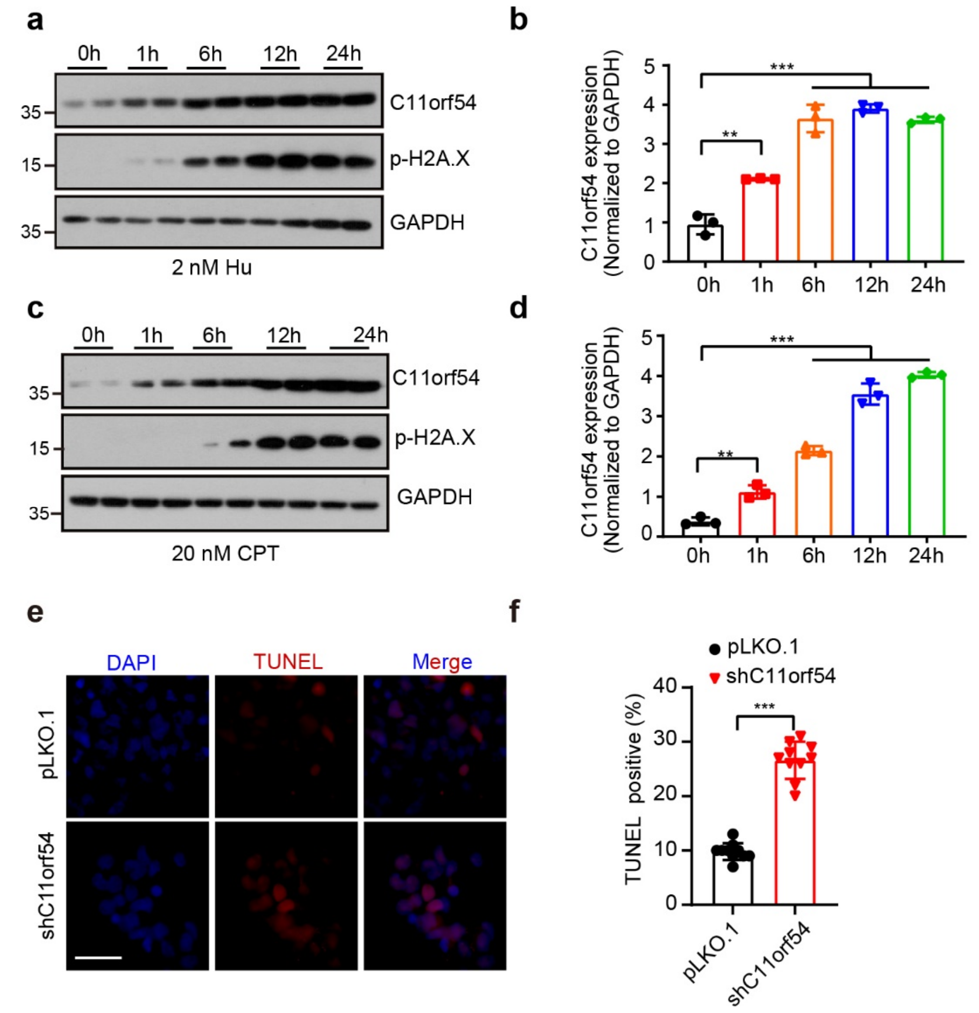

Supplementary Figure 3. The impact on the expression of C11orf54 by Hydroxyurea (Hu) and Camptothecin (CPT) and TUNEL assay

a, b Western blot (A) and quantitative results (B) of C11orf54 expression in PLC/PRF/5 cells with 2 mM Hydroxyurea (Hu) for different time points (0 h, 1 h, 6 h, 12 h and 24 h) (n = 3 biological replicates).

c, d Western blot (C) and quantitative results (D) of C11orf54 expression in PLC/PRF/5 cells with 20 nM Camptothecin (CPT) for different time points (0 h, 1 h, 6 h, 12 h and 24 h) (n = 3 biological replicates).

e, f Representative images of TUNEL assay (E) and quantitative results (F) in pLKO.1 and C11orf54 knockdown cells after 48hr 20μM Cisplatin treatment.

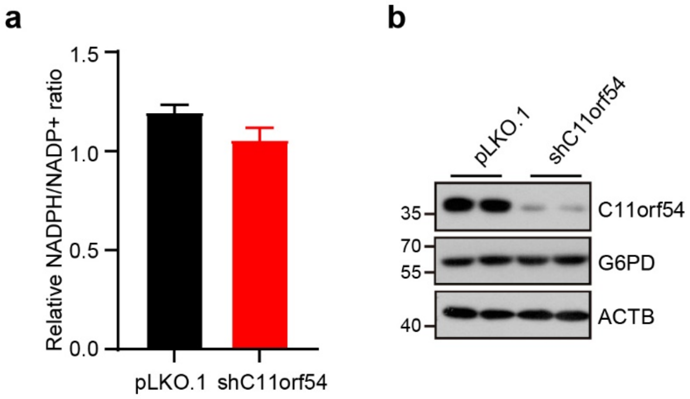

Supplementary Figure 4. The NADPH/NADP^{+} ratio and G6PD expression between C11orf54 knockdown and control cell

a Relative NADPH/NADP+ ratio in control and C11orf54 knockdown cells (n = 3 biological replicates).

b Western blot of the indicated proteins in control and C11orf54 knockdown cells (n = 3 biological replicates).

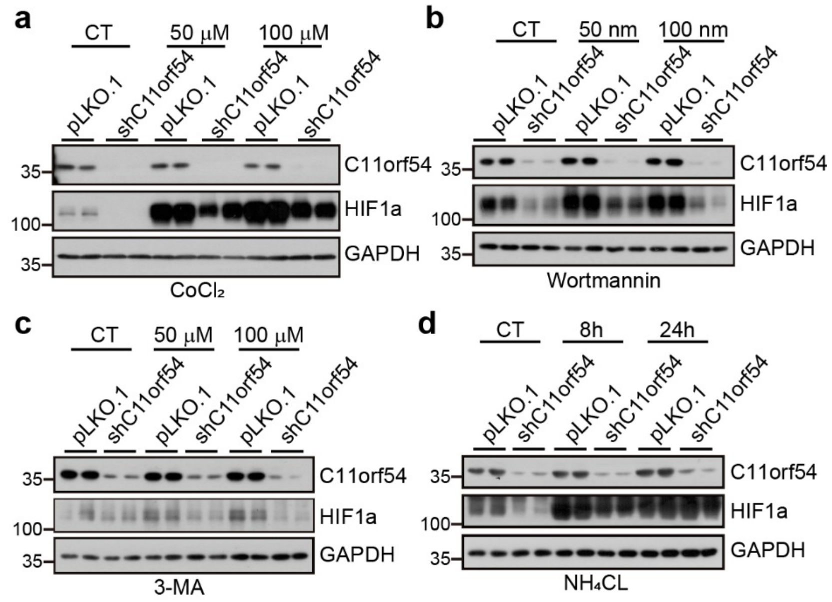

Supplementary Figure 5. The expression of HIF1A under Cocl$_2$ and autophagy inhibitors treatment between C11orf54 knockdown and control cell

a Western blot of the indicated proteins in control and C11orf54 knockdown cells upon 50 μM and 100 μM CoCl$_2$ treatment for 12 h.

b Western blots of the indicated proteins in control and C11orf54 knockdown cells upon 50 μM and 100 μM 3-Methyladnine (3-MA) treatment for 12 h.

c Western blot of the indicated proteins in control and C11orf54 knockdown cells upon 10 mM NH$_4$Cl treatment for 8 h and 24 h.

d Western blot of the indicated proteins in control and C11orf54 knockdown cells upon 50 nM and 100 nM Wortmannin treatment for 12 h.

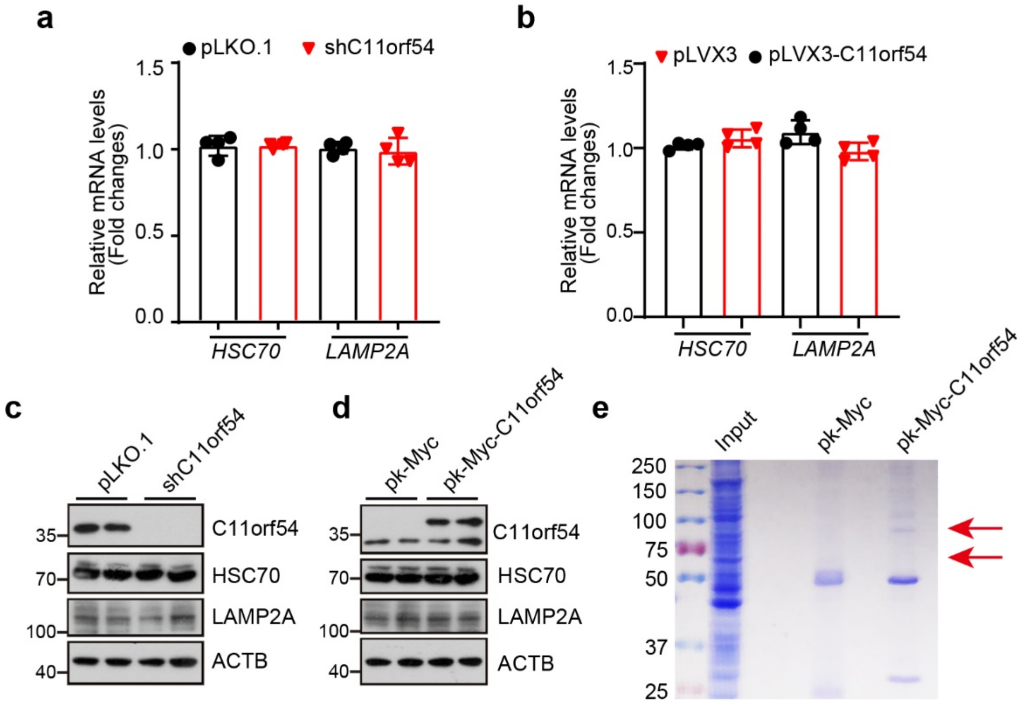

Supplementary Figure 6. The expression of HSC70 and LAMP2A in C11orf54 knockdown and overexpression cells

a Q-PCR experiment analysis of the mRNA expression of HSC70 and LAMP2A in C11orf54 knockdown and control cells. (n = 4 biological replicates, data are presented as mean values ± SD).

b Q-PCR experiment analysis of the mRNA expression of HSC70 and LAMP2A in C11orf54 overexpression and control cells. (n = 4 biological replicates, data are presented as mean values ± SD).

c Western blot of HSC70 and LAMP2A in C11orf54 knockdown and control cells.

d Western blot of HSC70 and LAMP2A in C11orf54 overexpression and control cells.

e Identification of C11orf54 binding proteins using co-immunoprecipitation (co-ip)-coupled liquid chromatography tandem mass spectrometry (LC-MS/MS)-

based interactome analysis. The arrows indicated intended gels for mass spectrometry.

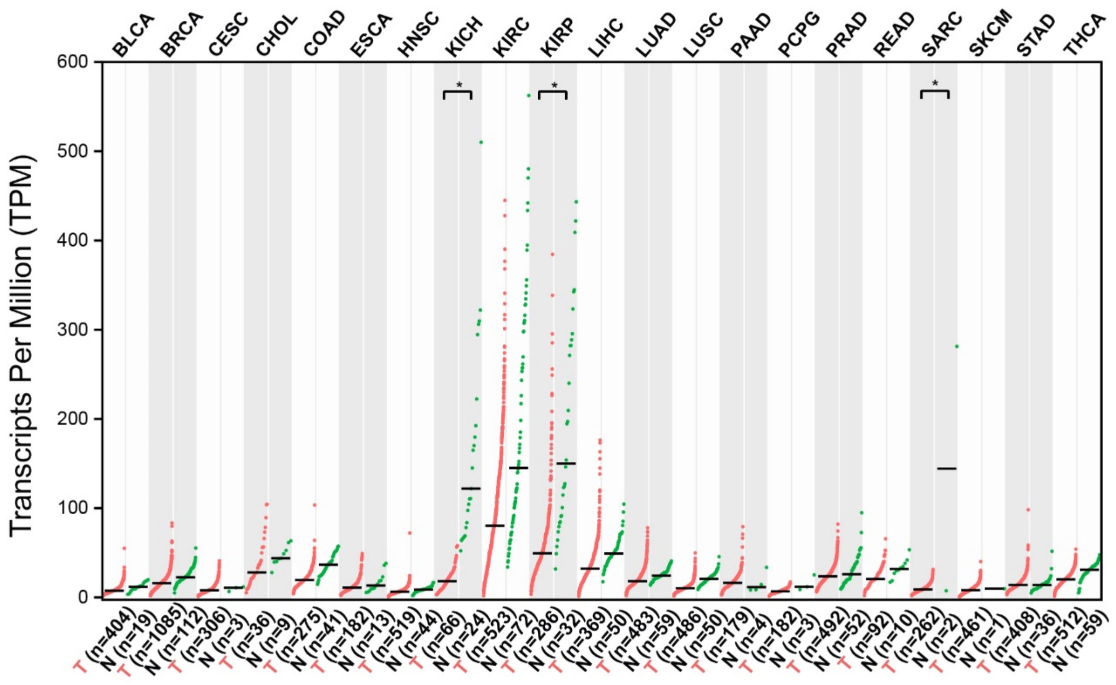

Supplementary Figure 7. The expression of C11orf54 in several cancer tissue samples based on TCGA data from the GEPIA website (http://gepia.cancer-pku.cn/)

Supplementary Figure 8. The FACS gating strategy.

The example of the FACS gating strategy in HR assay (Fig.4e) is shown. The proportion of total cells were defined as shown in the left panel. The proportion of cells with HR were defined as shown in the right panel.

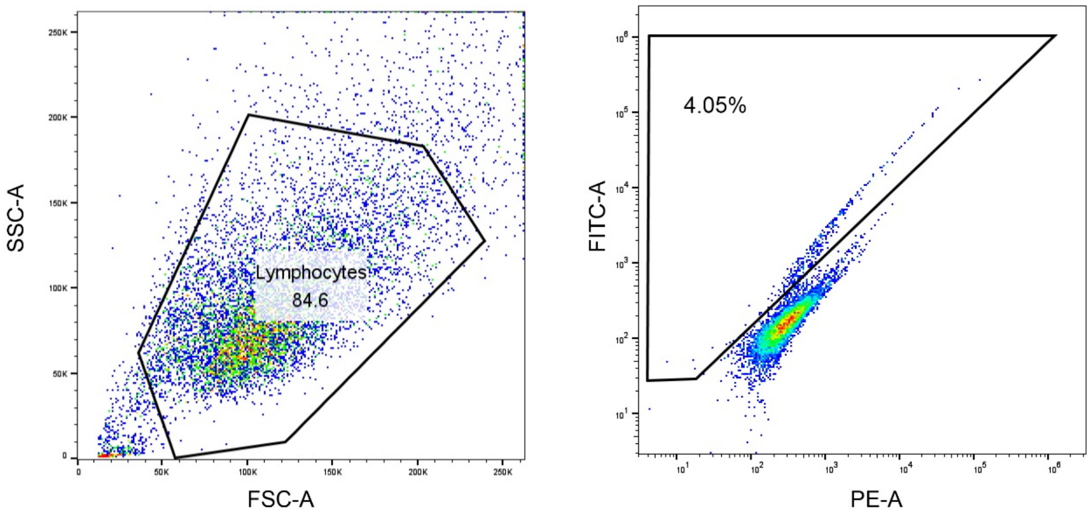

Supplementary Figure 9
Unedited/uncropped western blot gels

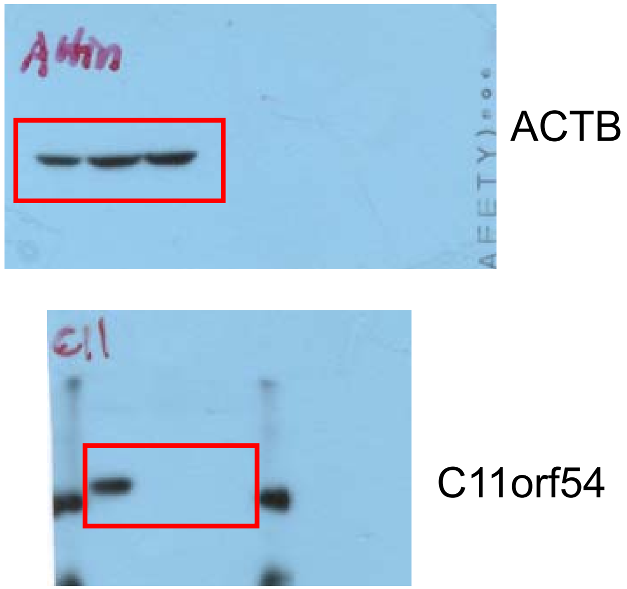

Fig. 1a

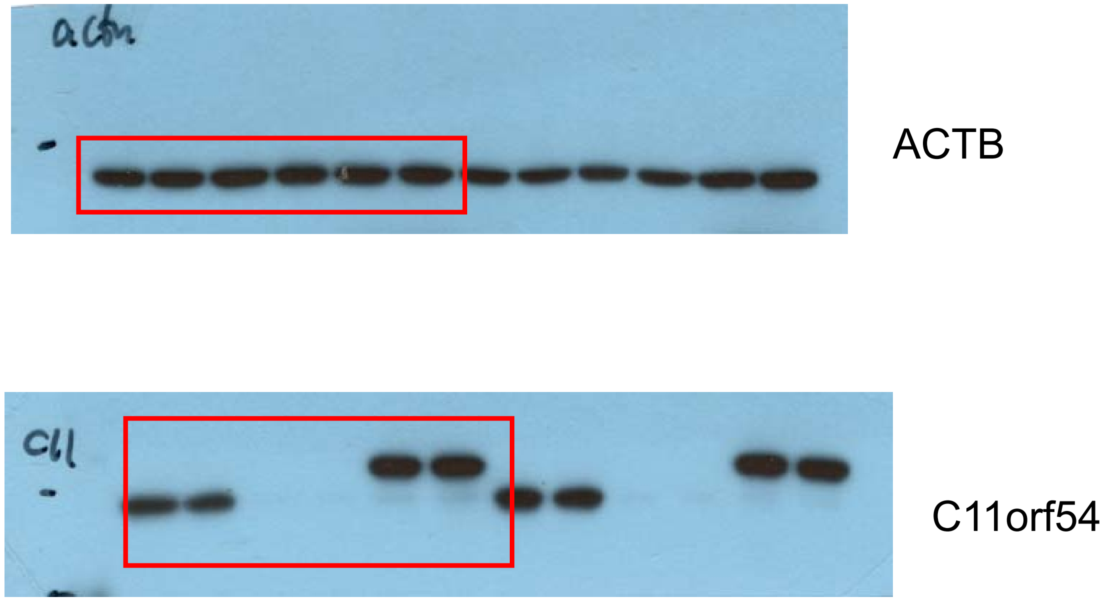

Fig. 1b

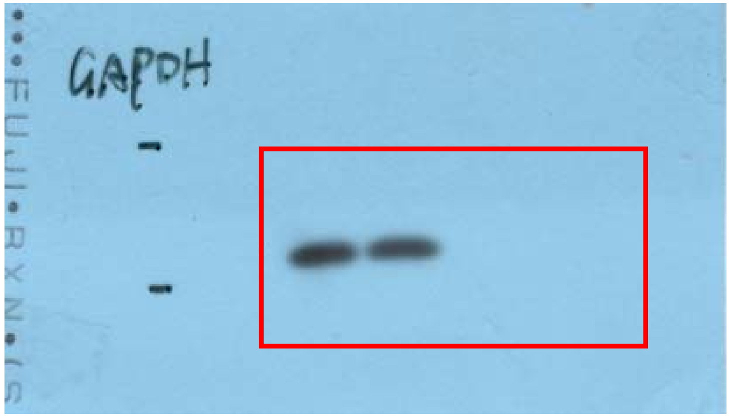

GAPDH

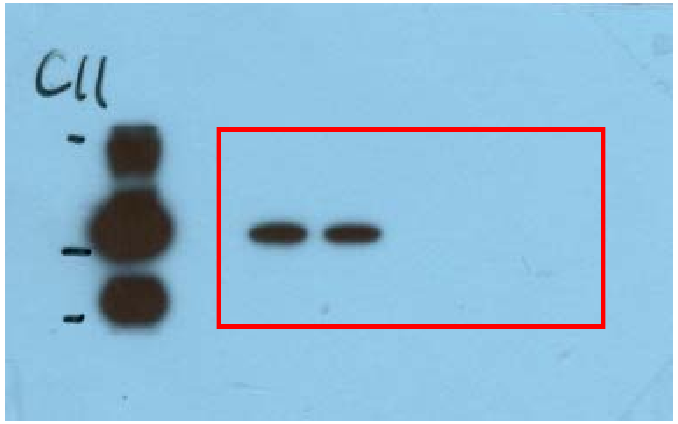

C11orf54

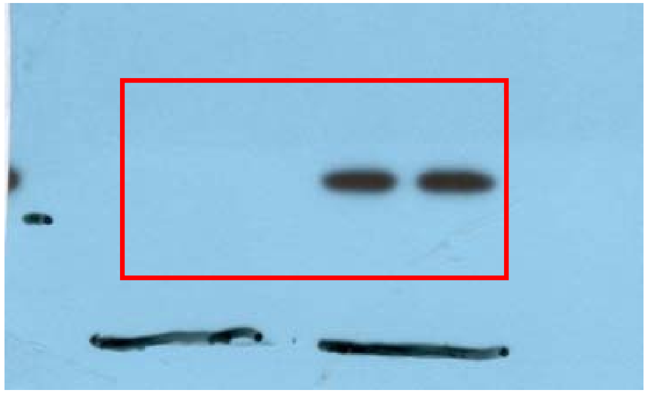

H3

Fig. 1f

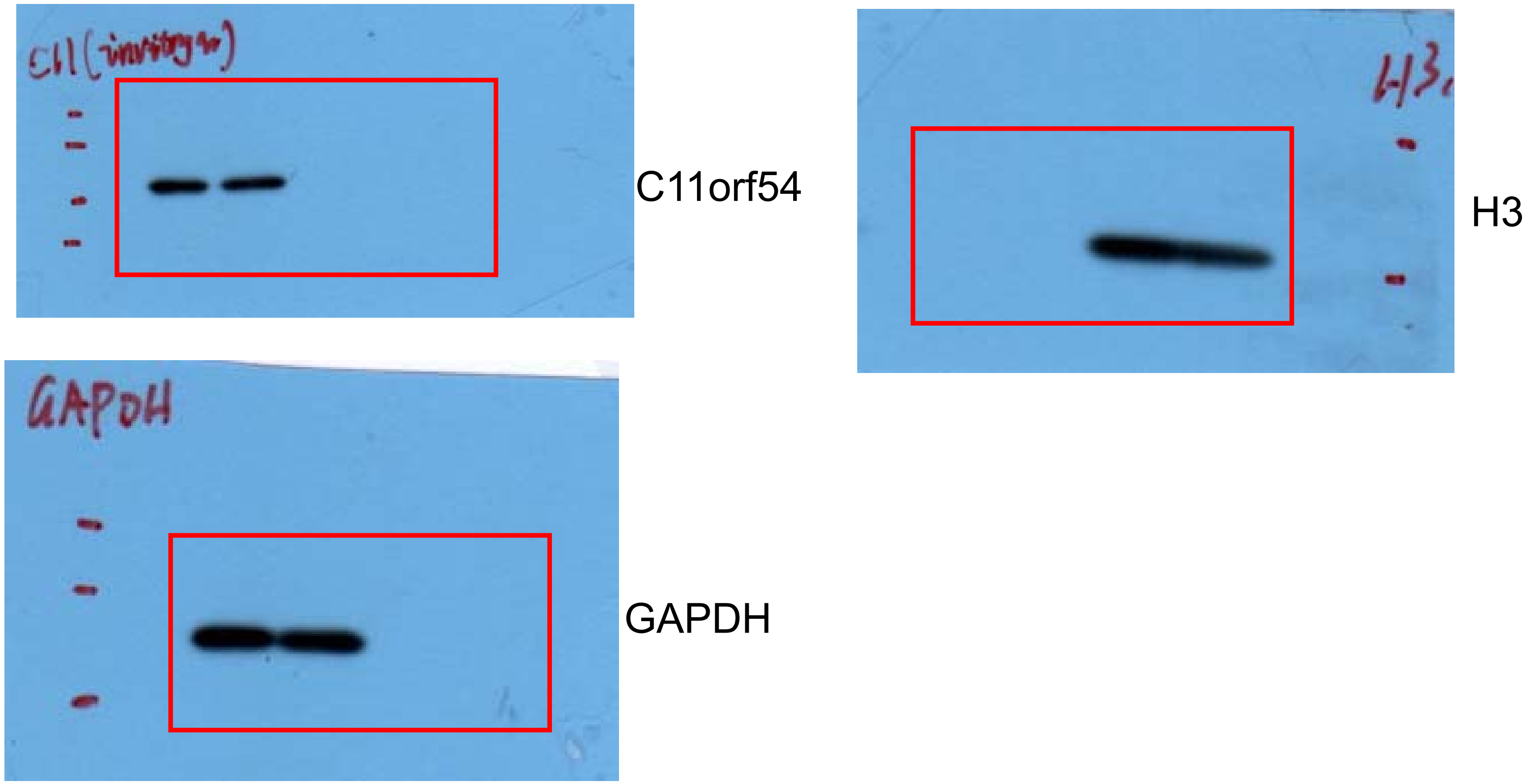

Fig. 1g

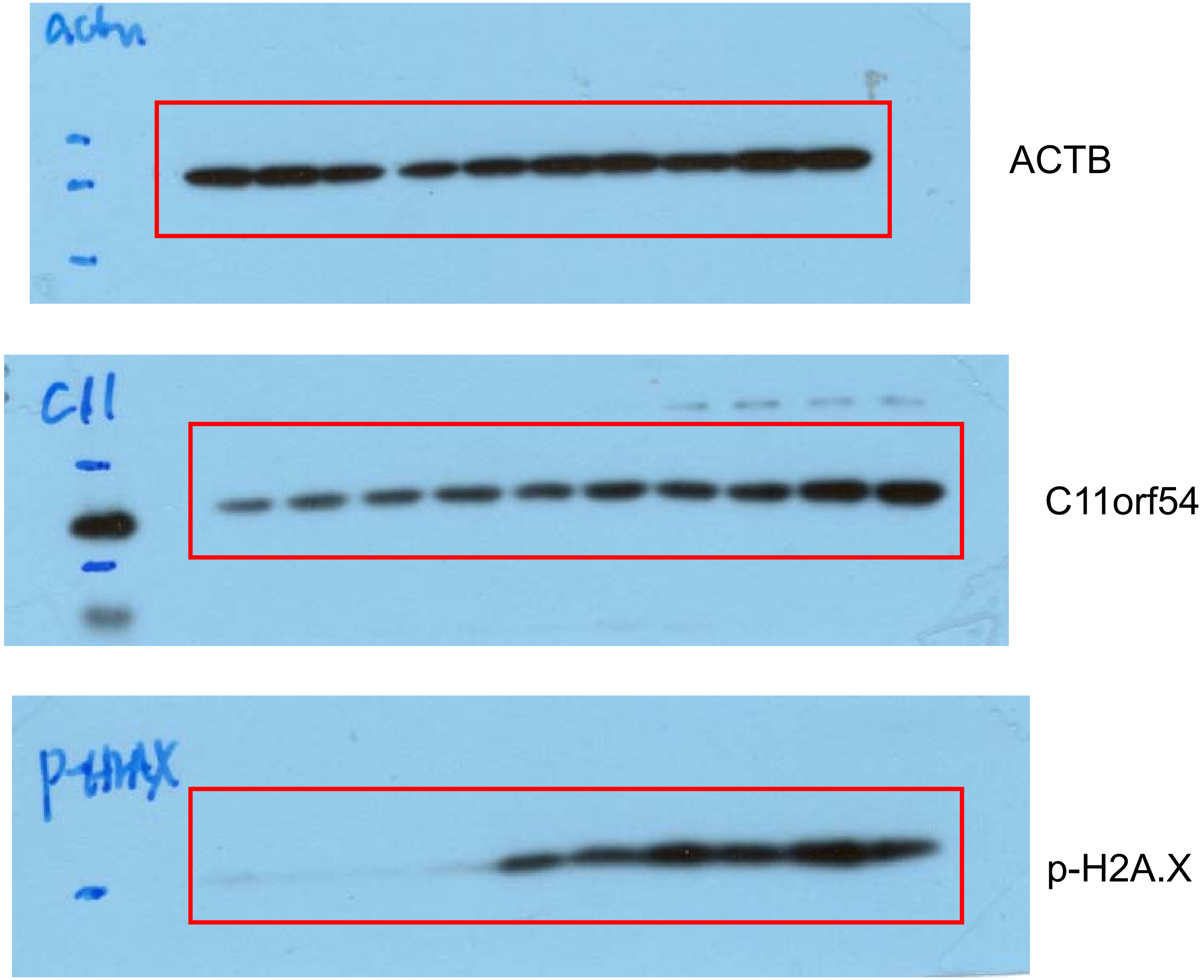

Fig. 2f

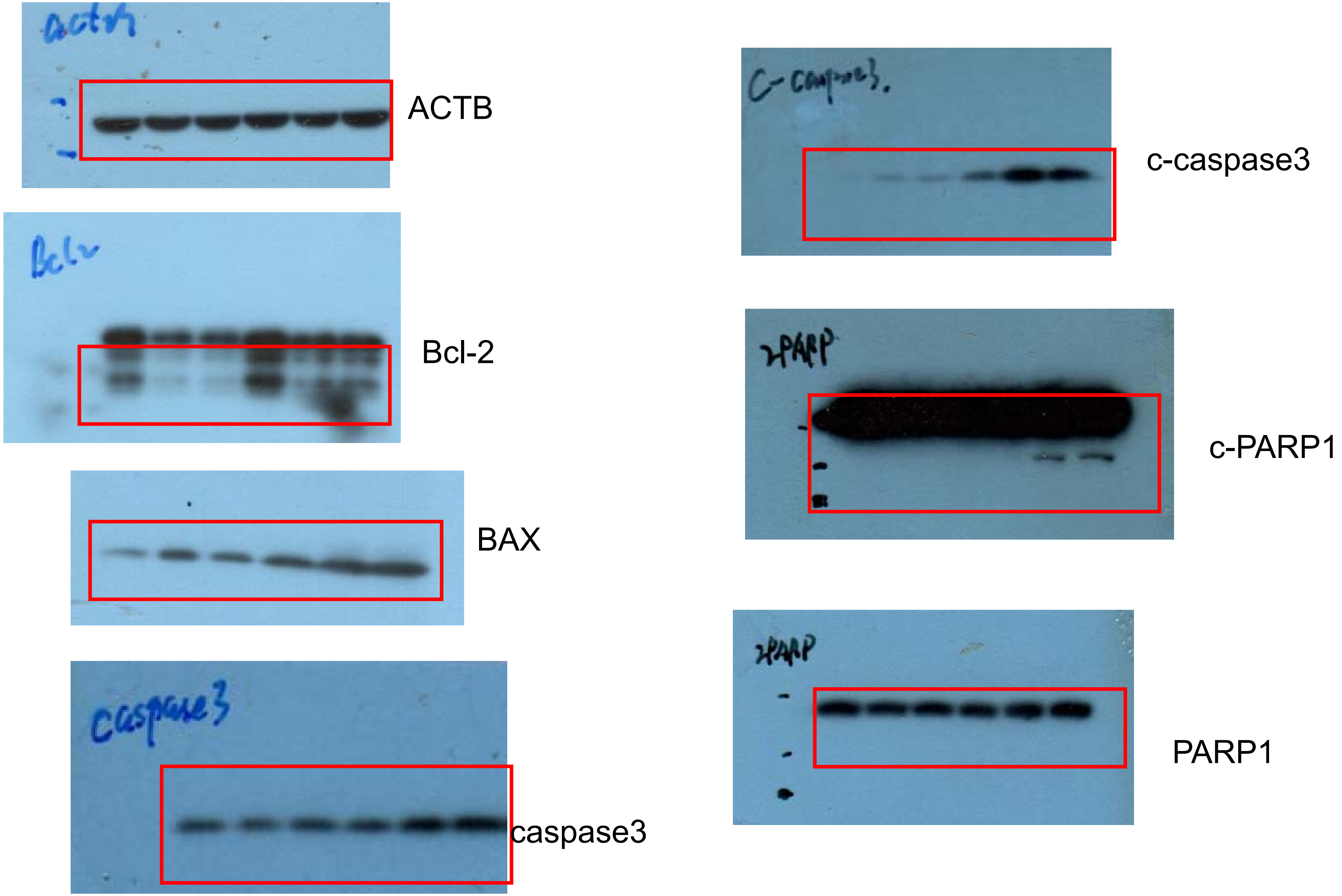

Fig. 2m

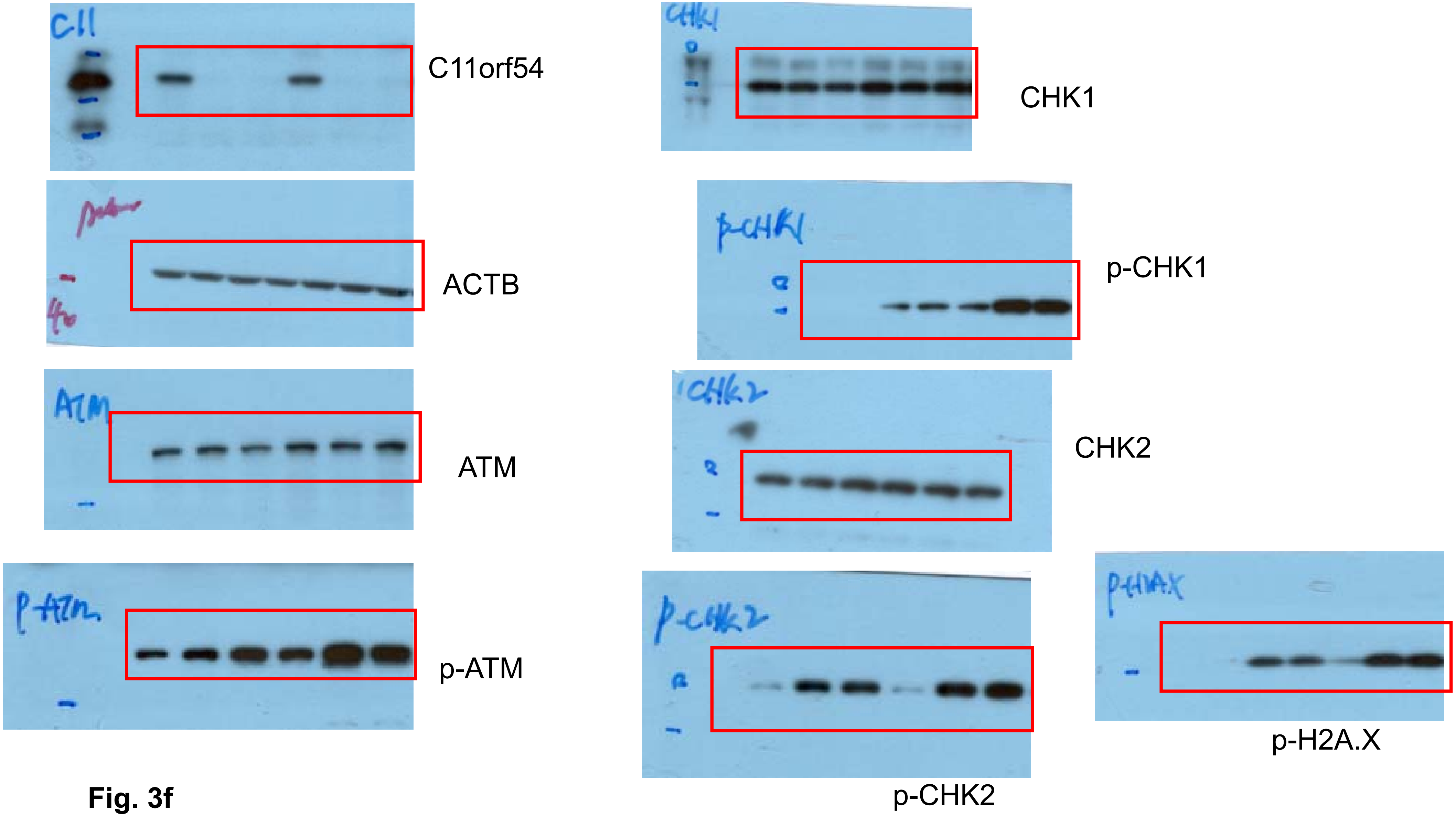

Fig. 3f

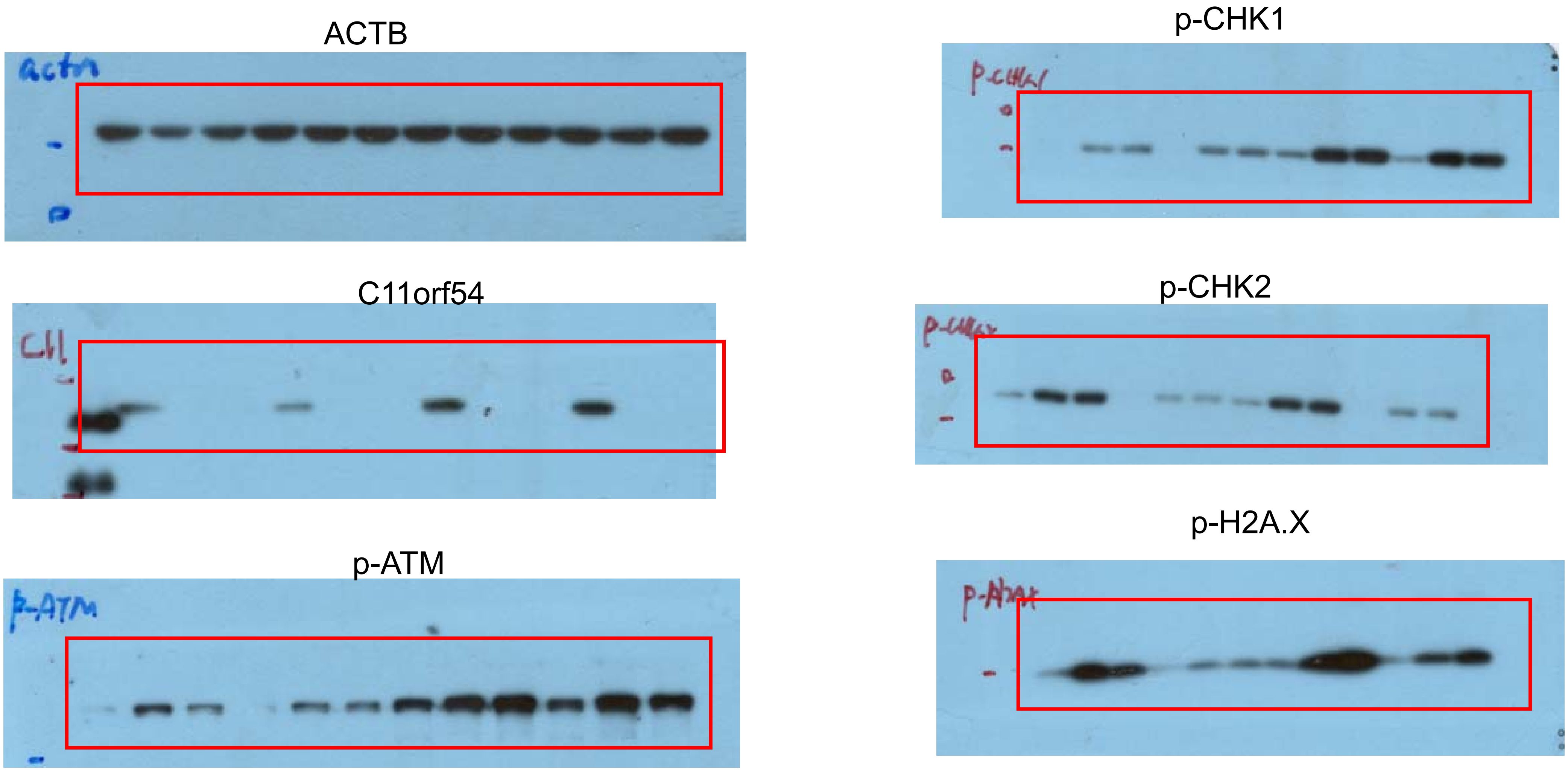

Fig. 3h

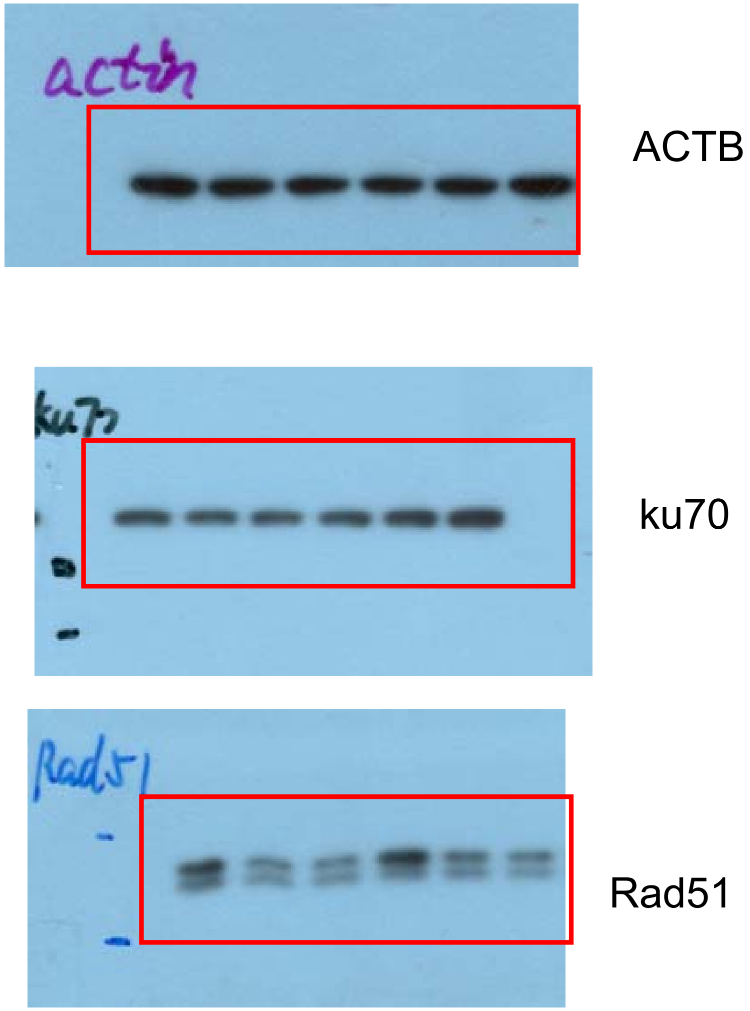

Fig. 4a

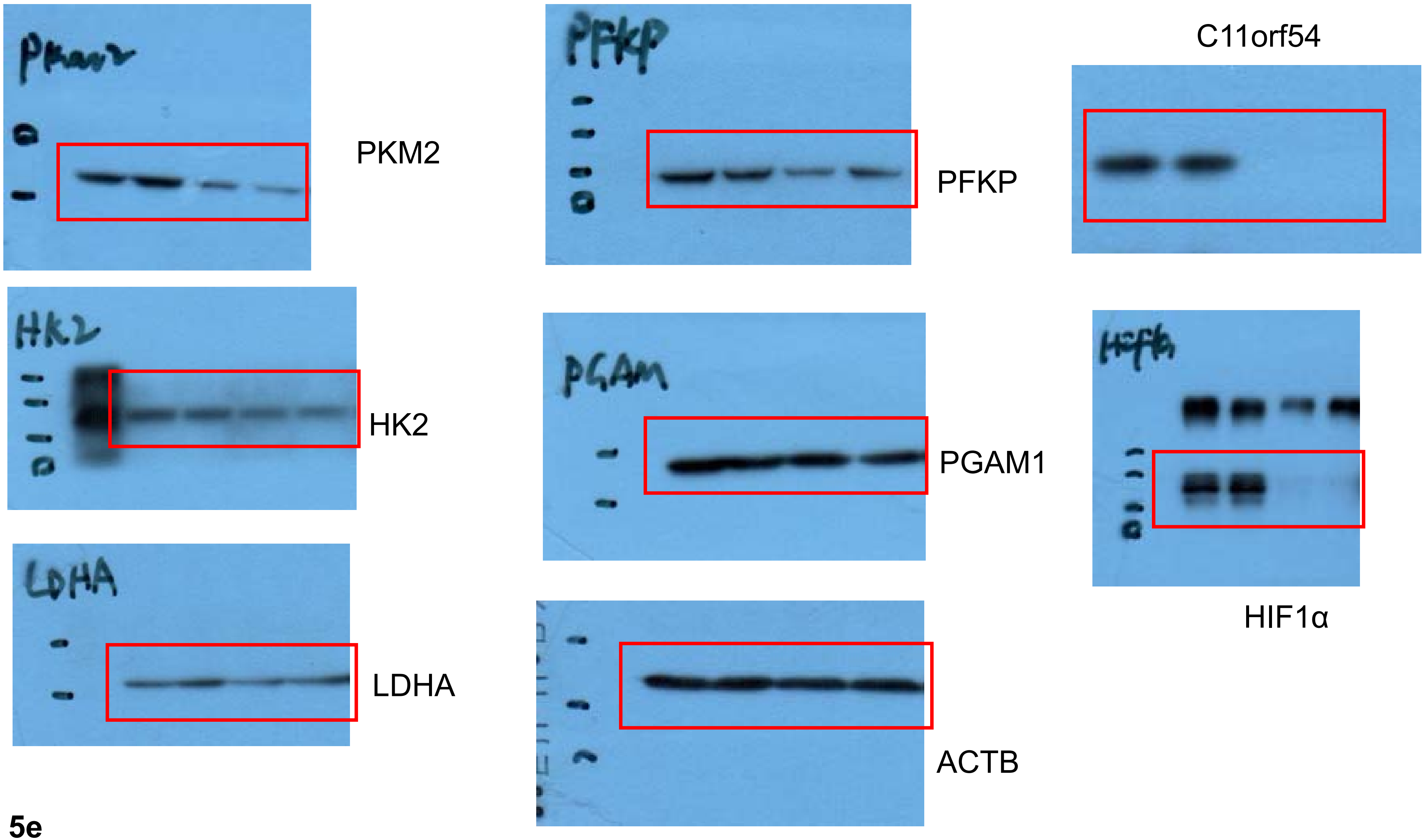

Fig. 5e

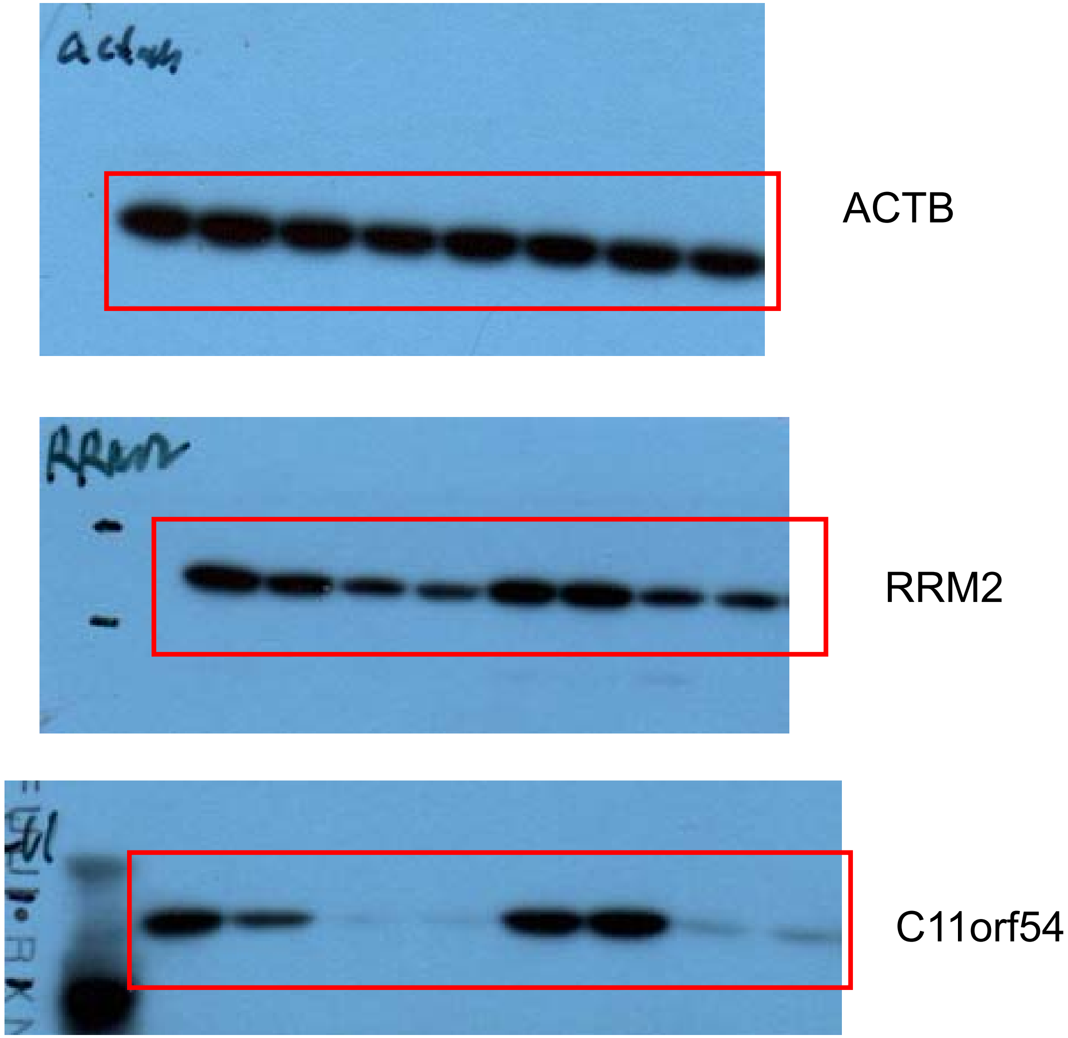

Fig. 6b

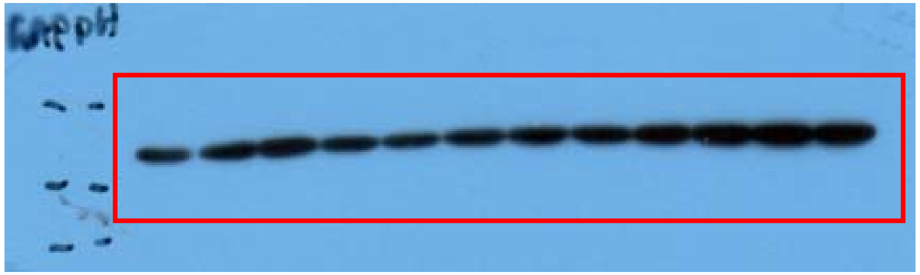

GAPDH

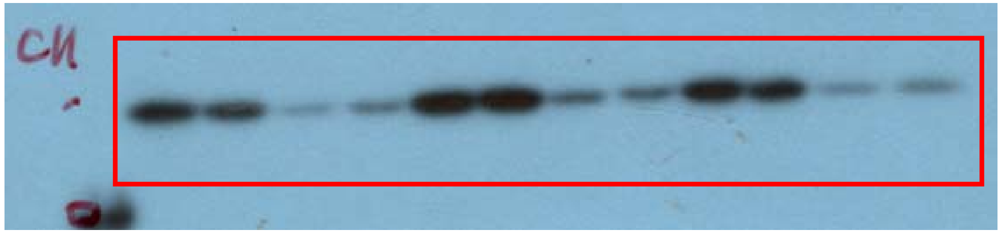

C11orf54

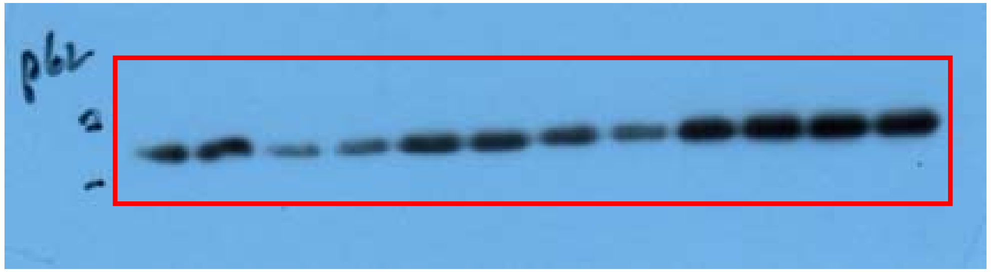

p62

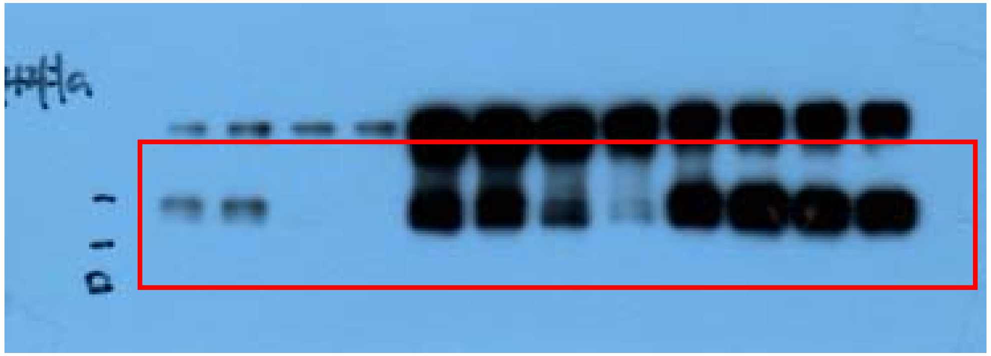

HIF1α

Fig. 7a

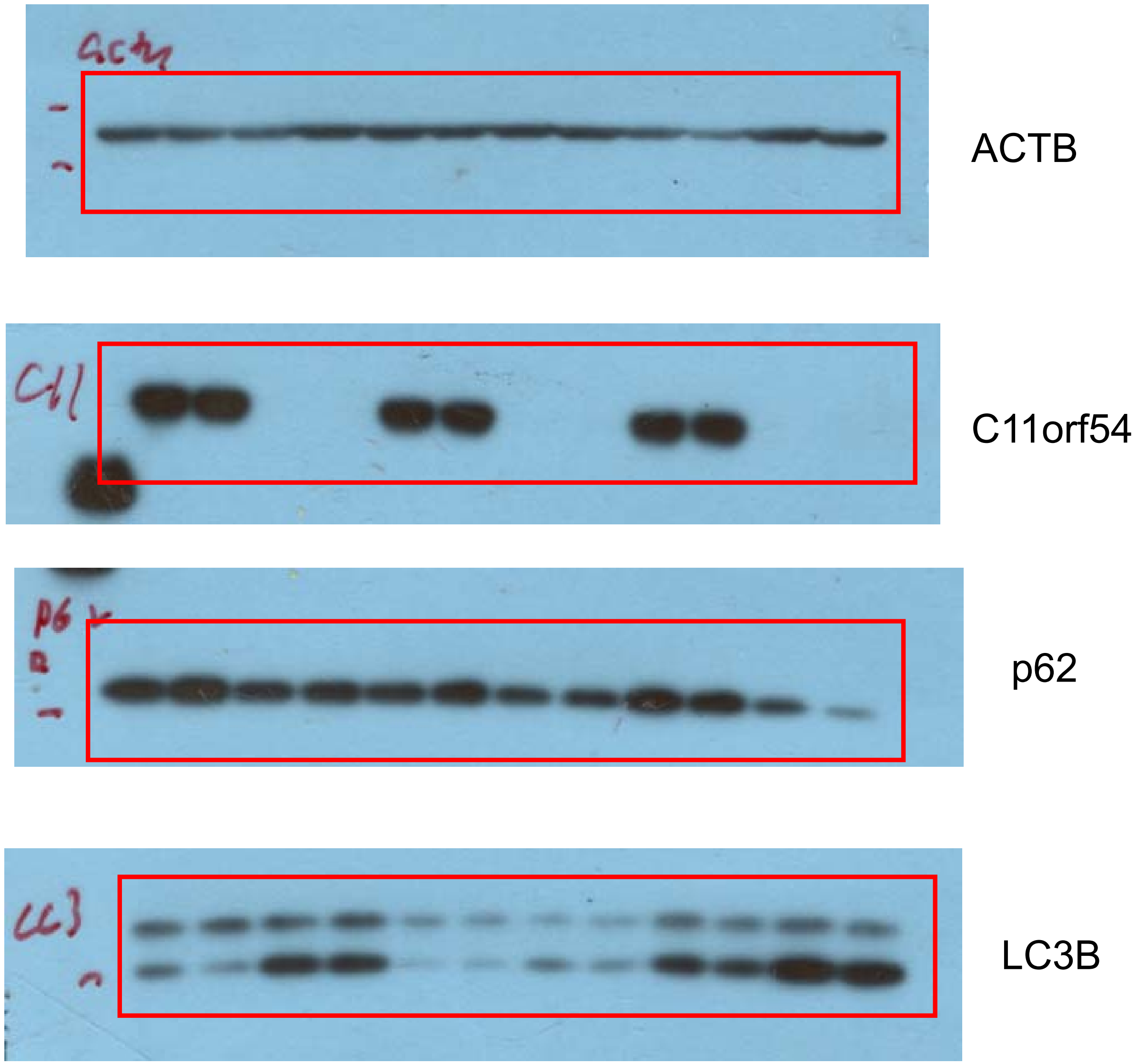

Fig. 7c

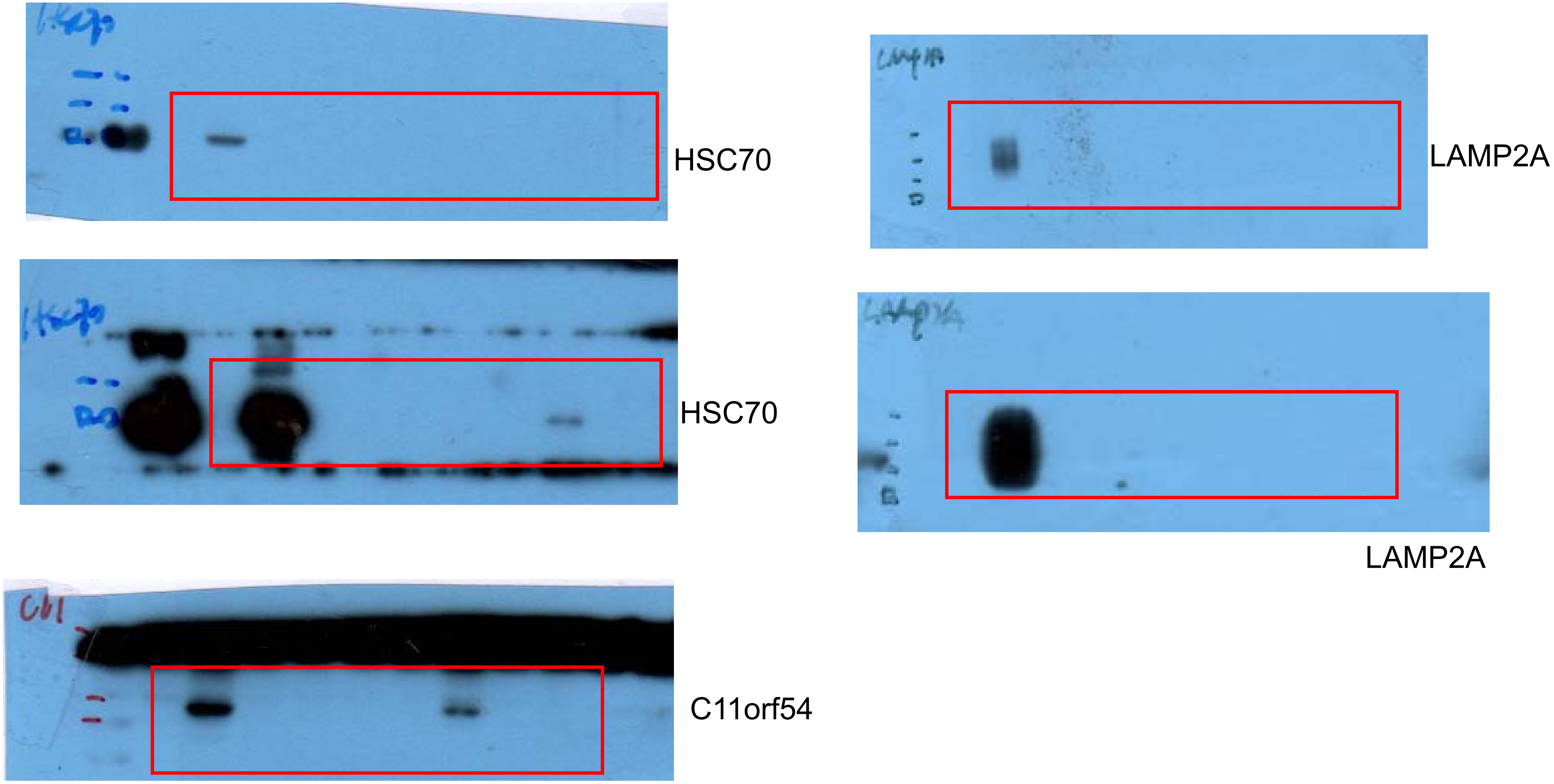

Fig. 8c

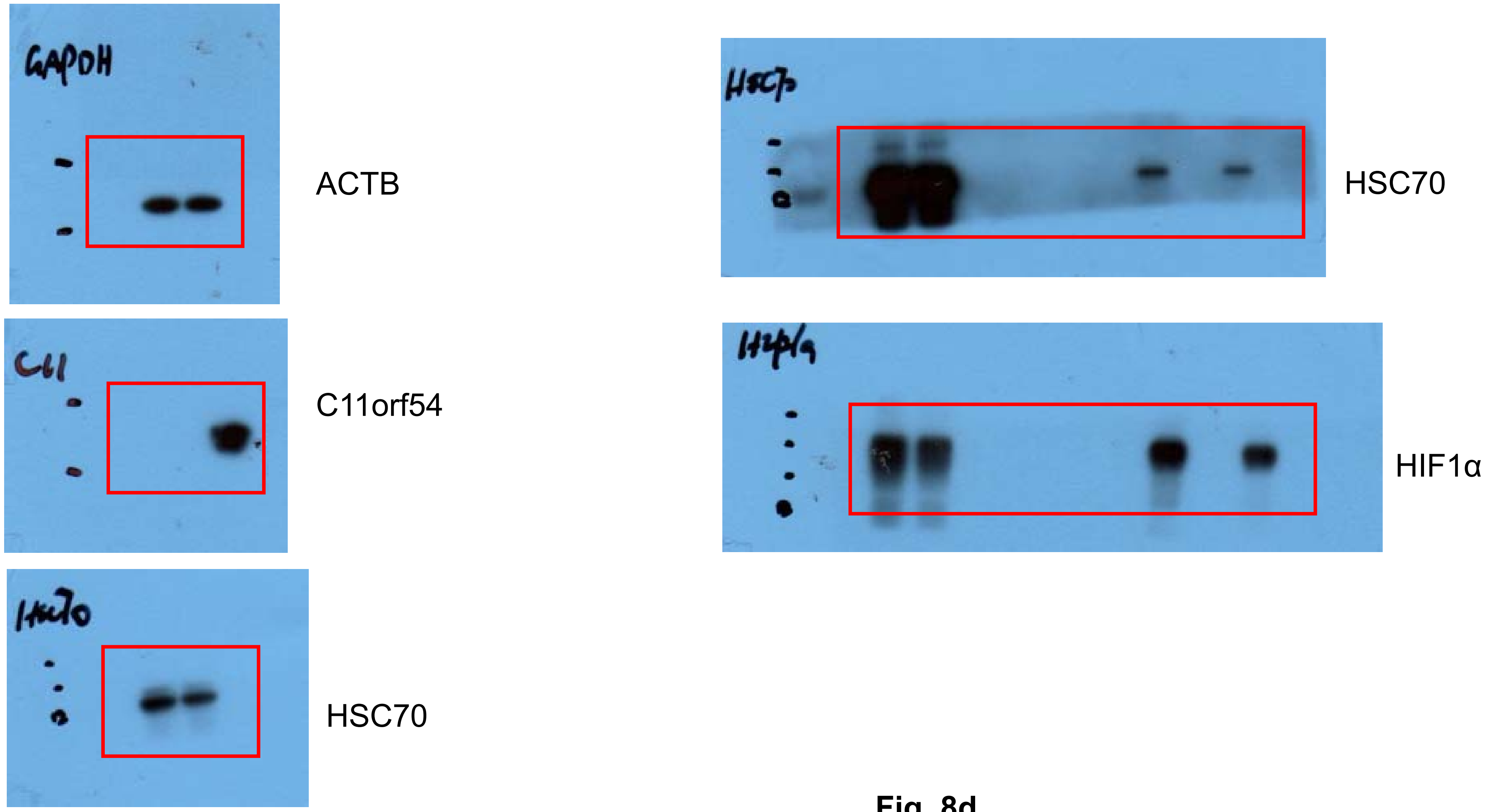

Fig. 8d

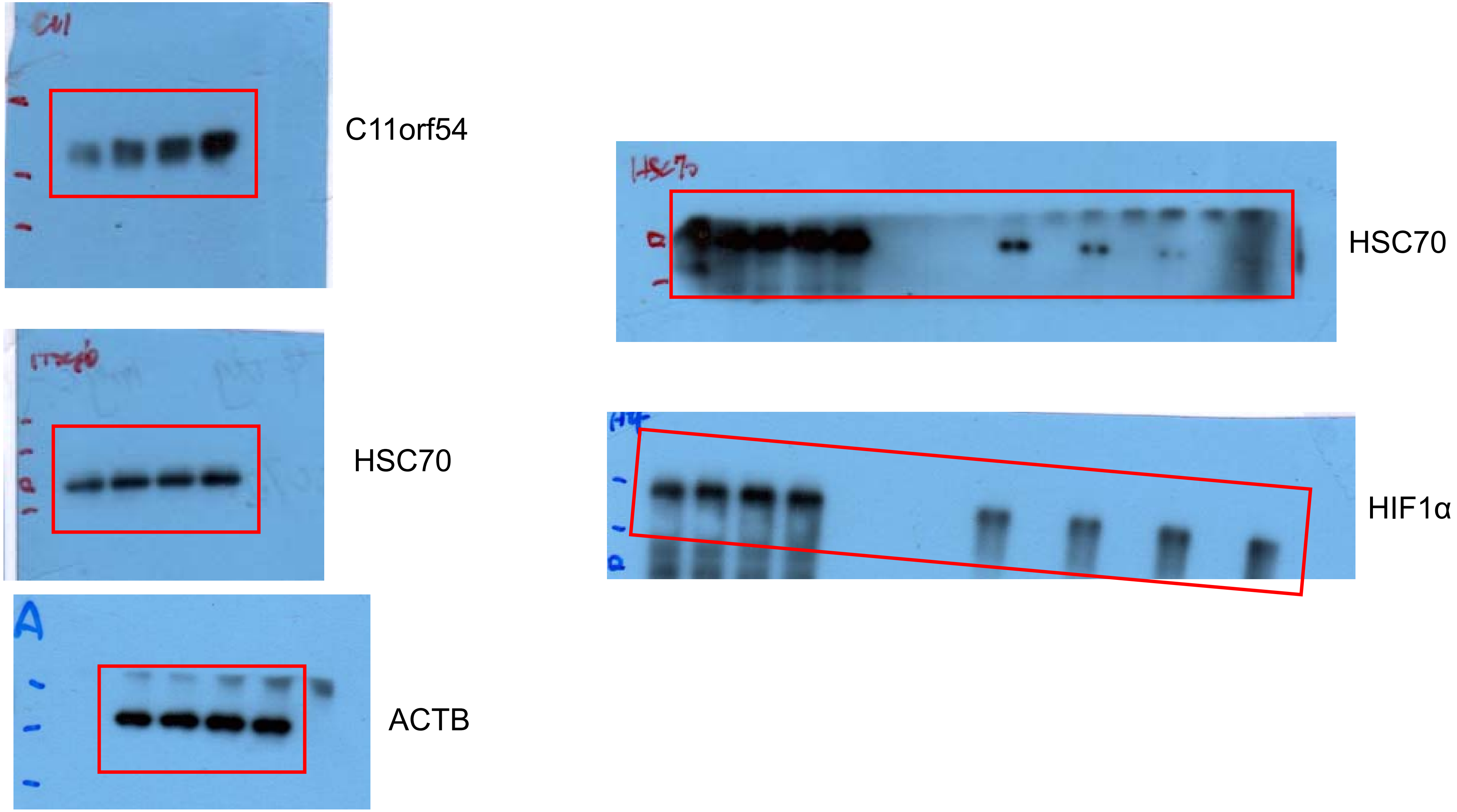

Fig. 8e

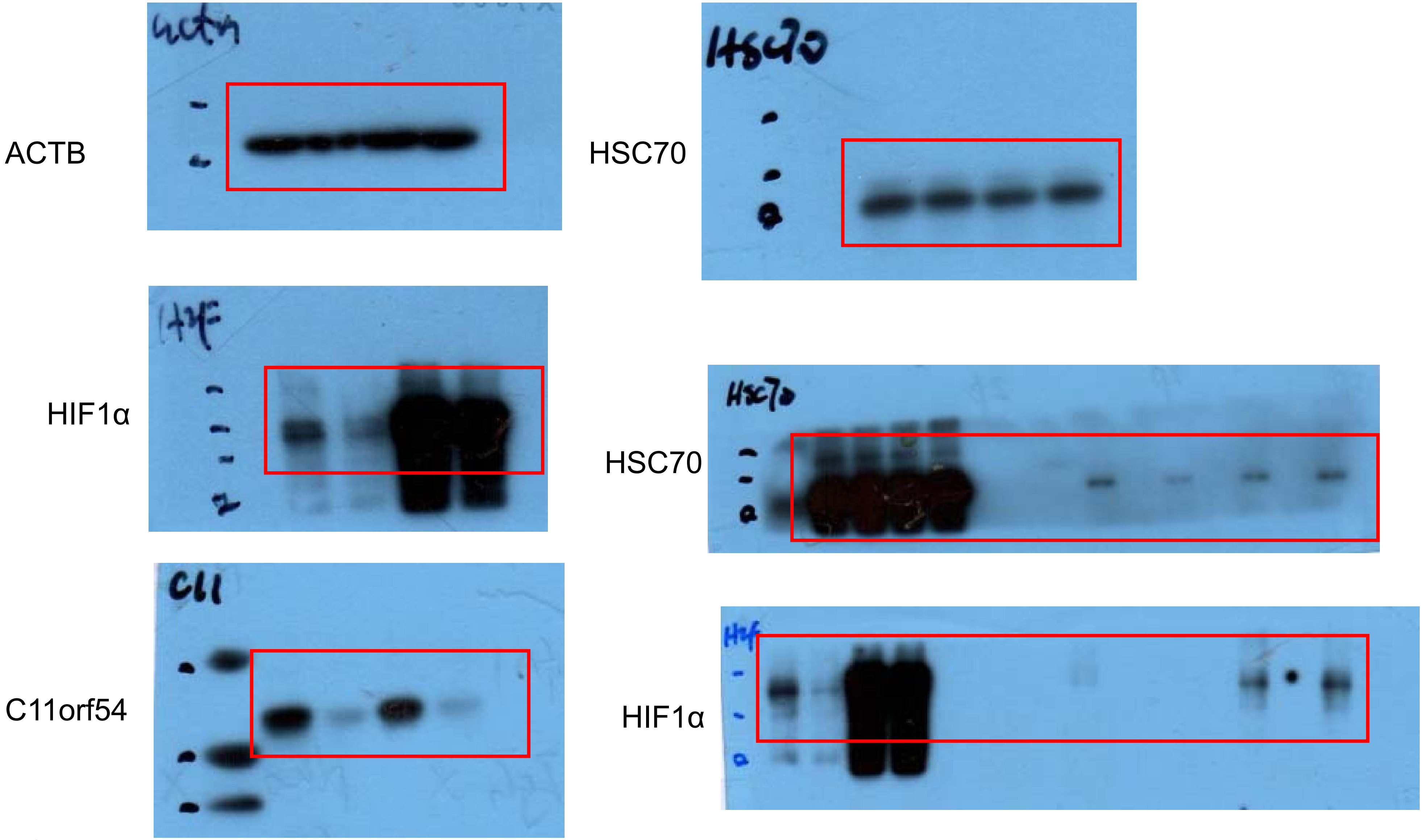

Fig. 8f

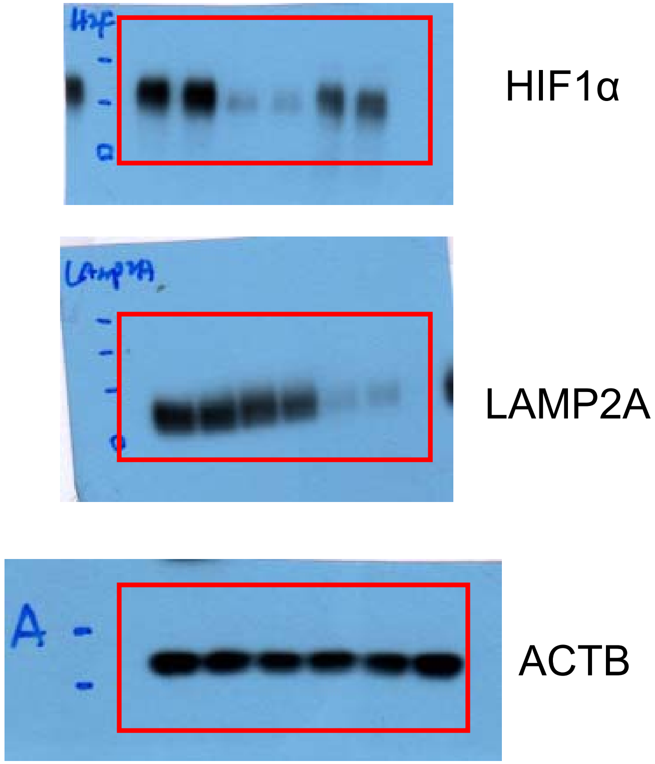

Fig. 8g

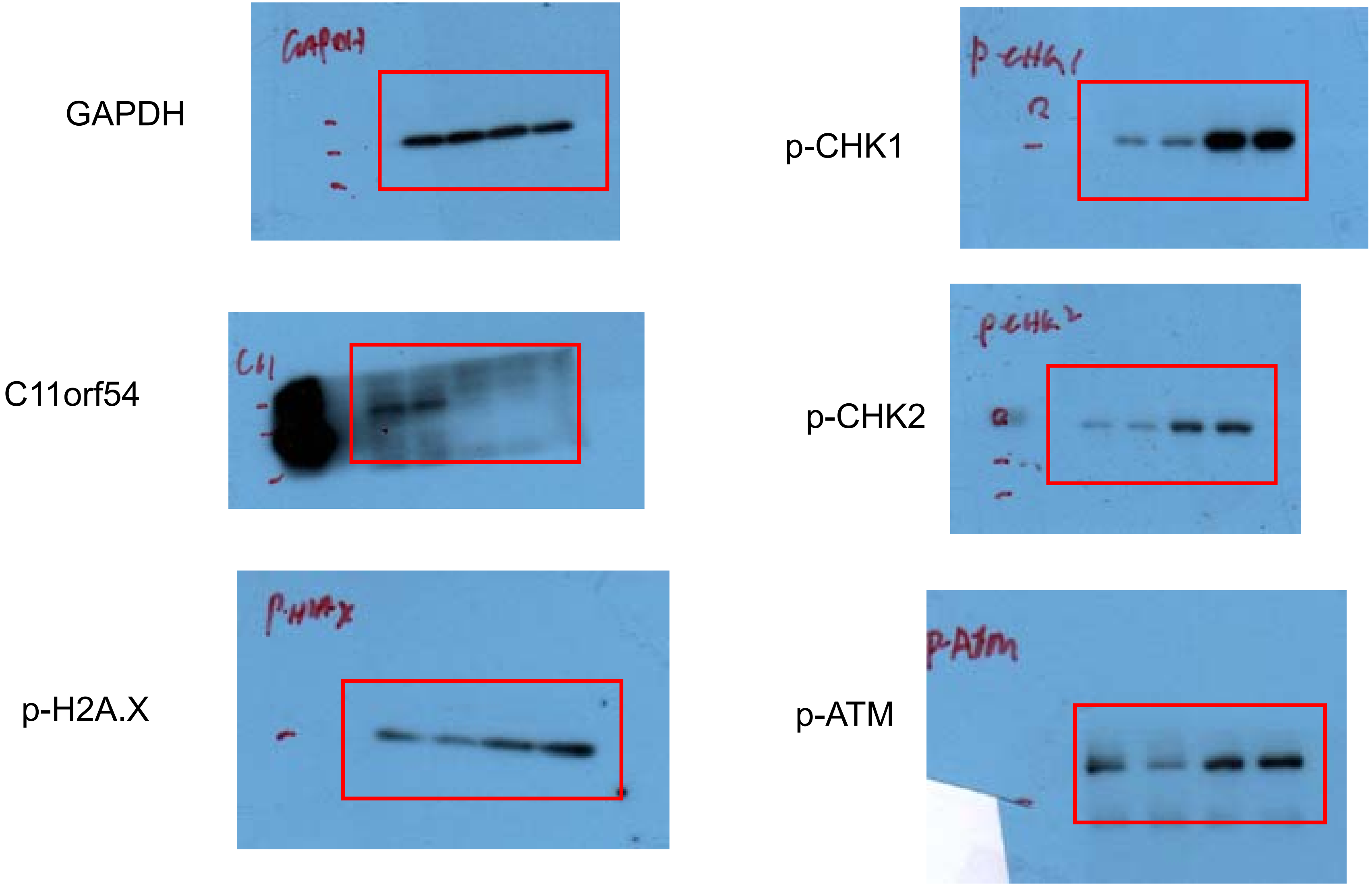

Supplementary Fig. 2d

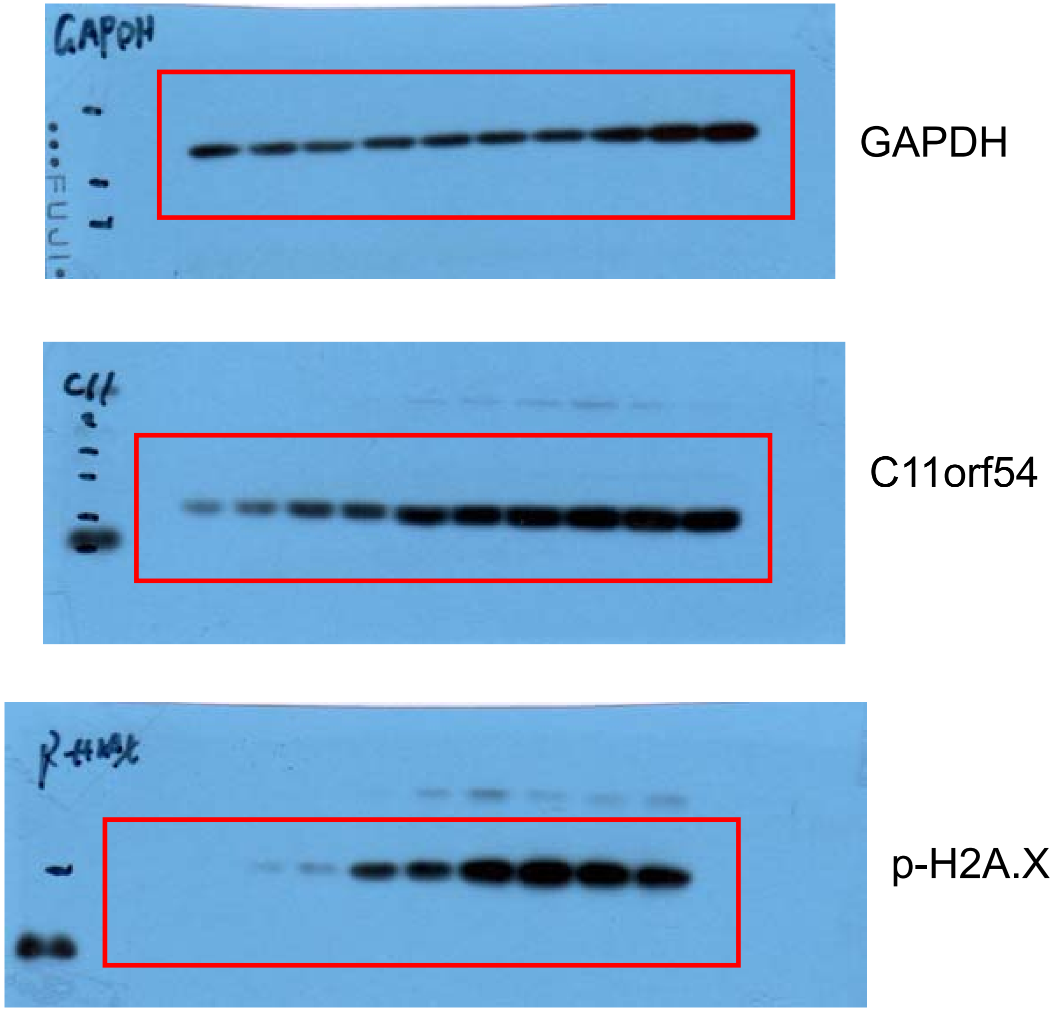

Supplementary Fig. 3a

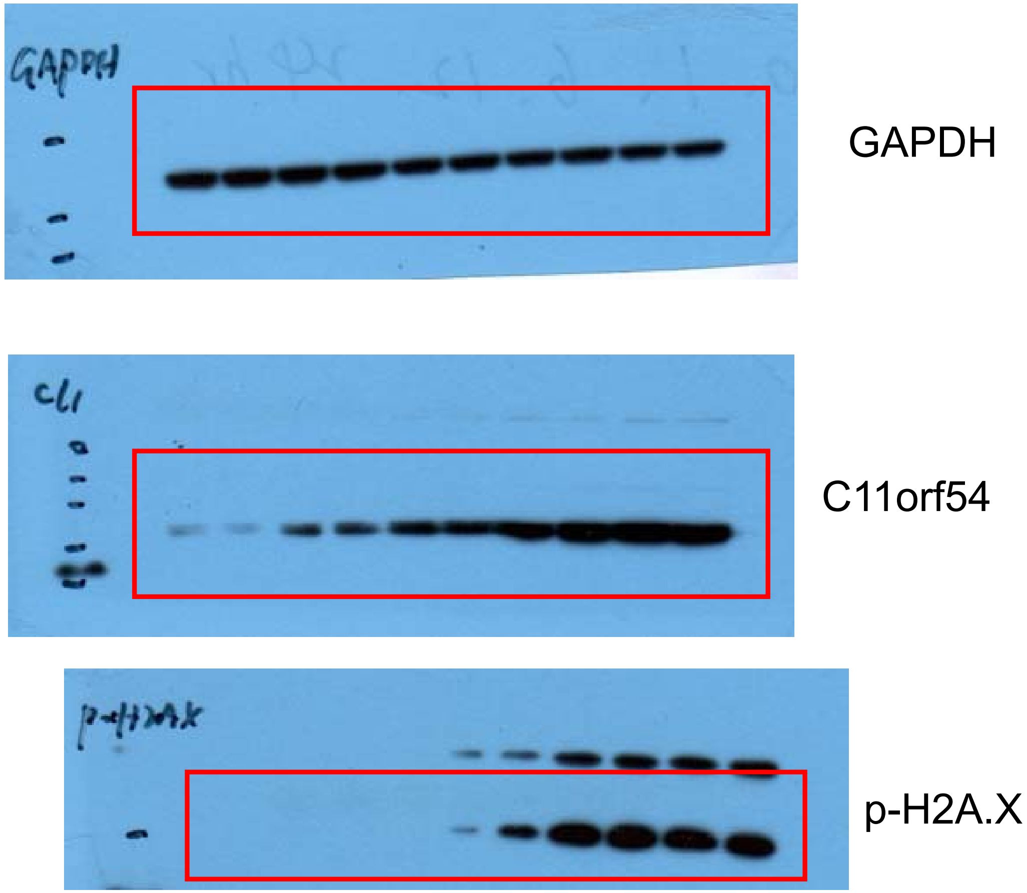

Supplementary Fig. 3c

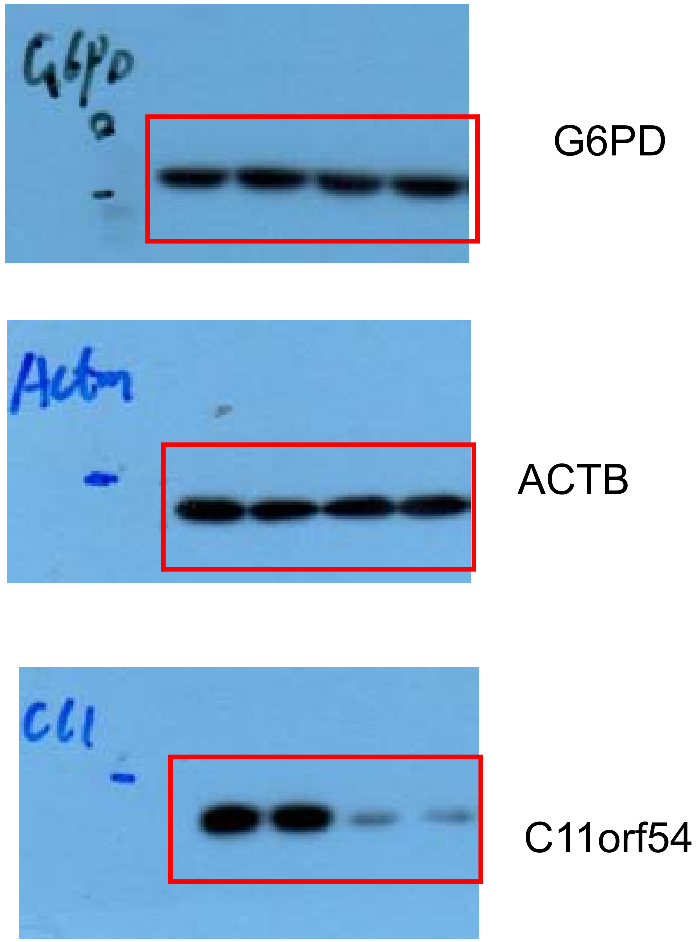

Supplementary Fig. 4b

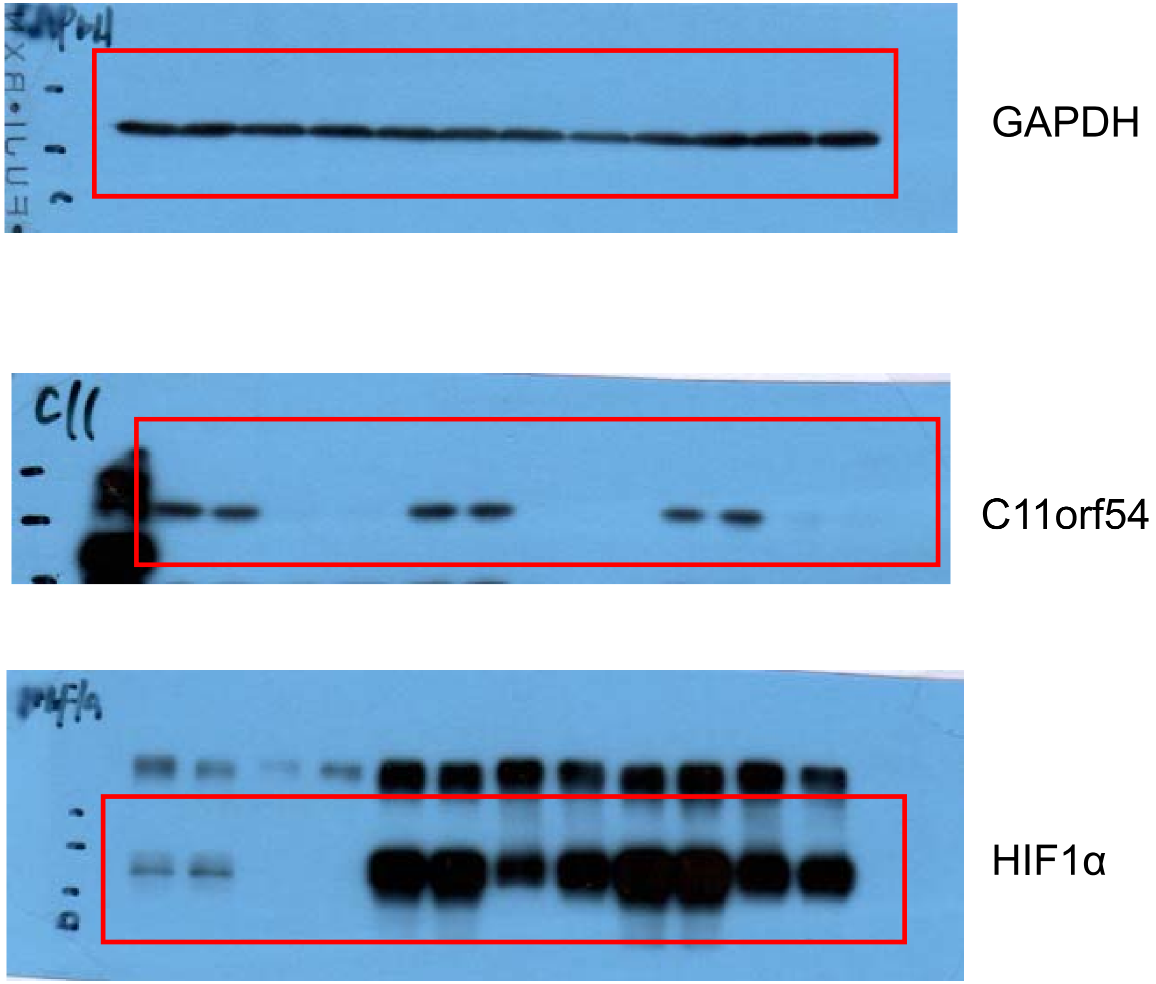

Supplementary Fig. 5a

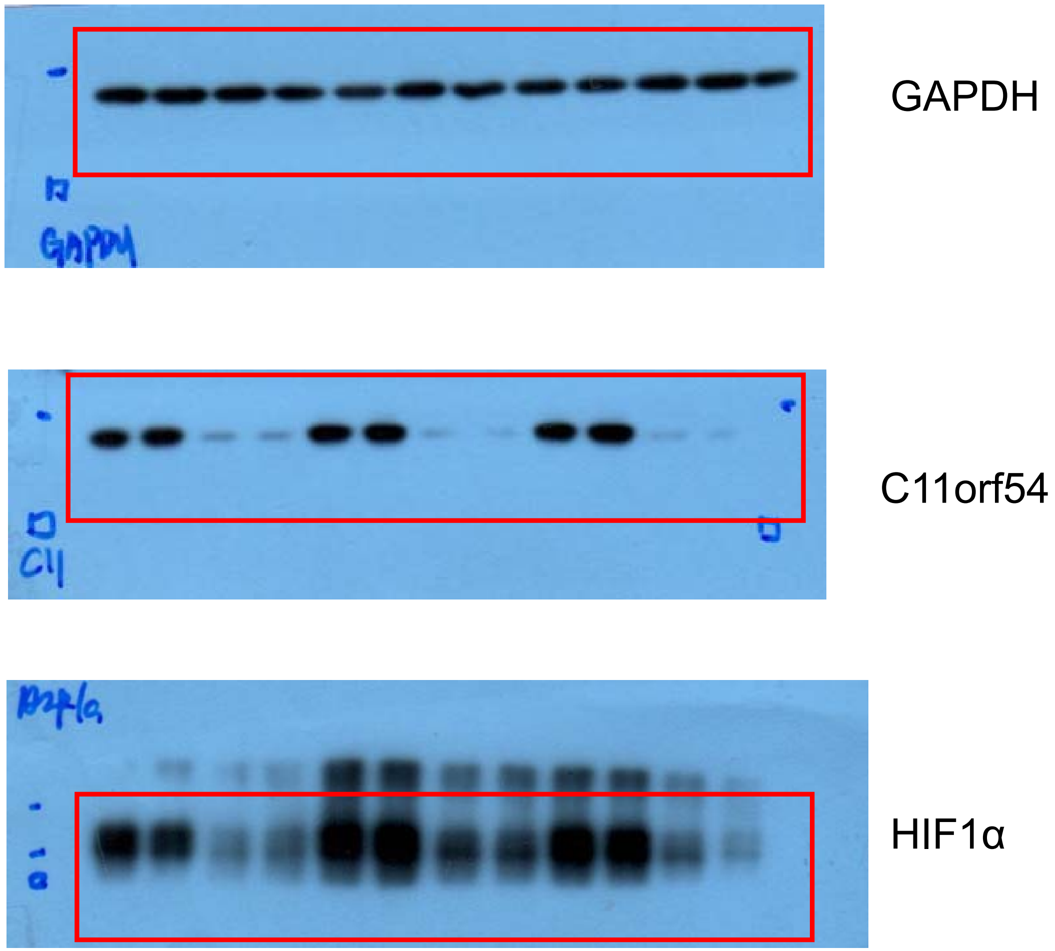

Supplementary Fig. 5b

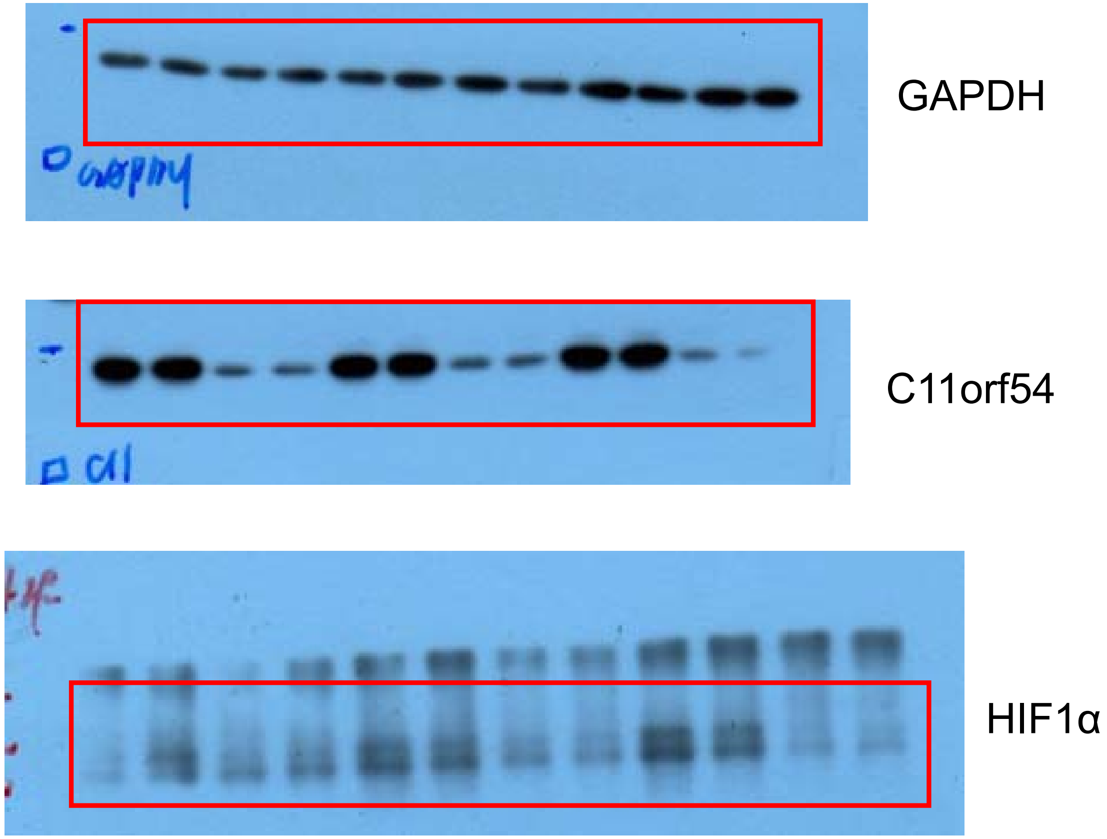

Supplementary Fig. 5c

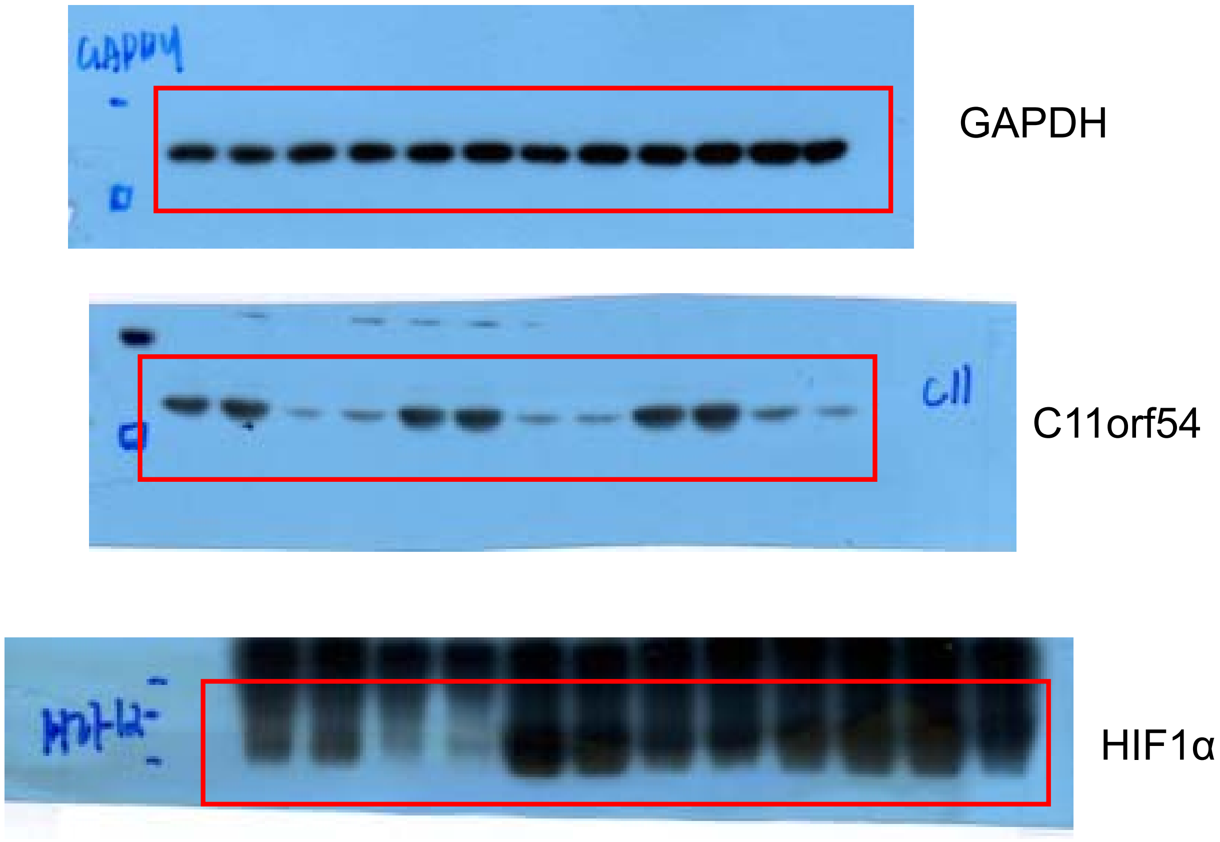

Supplementary Fig. 5d

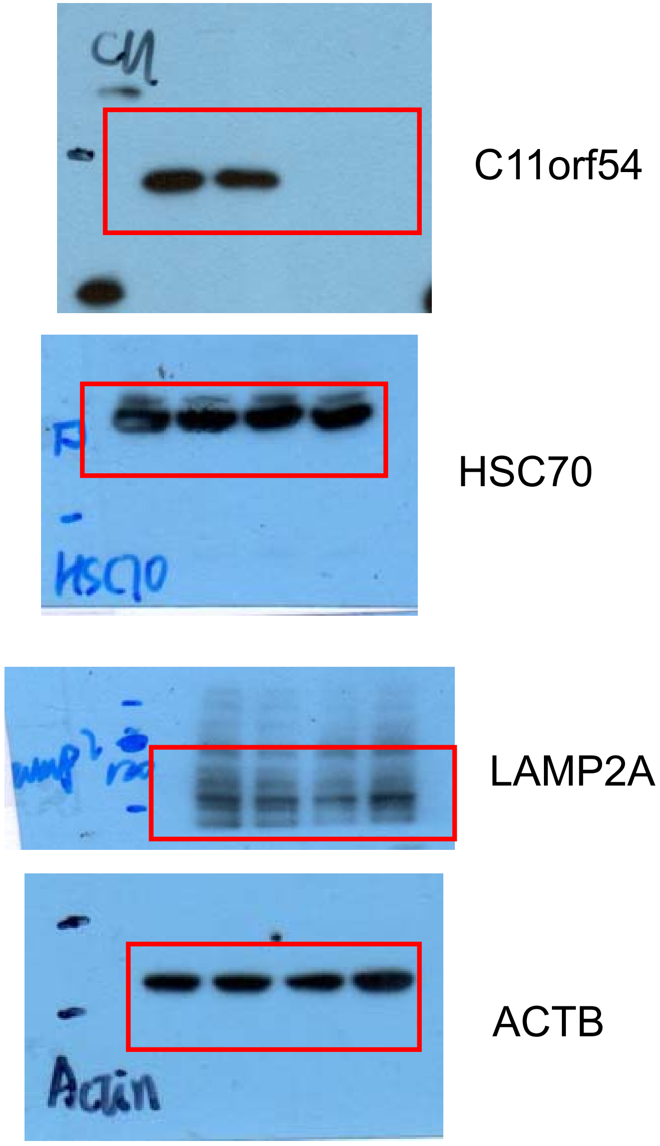

Supplementary Fig. 6c

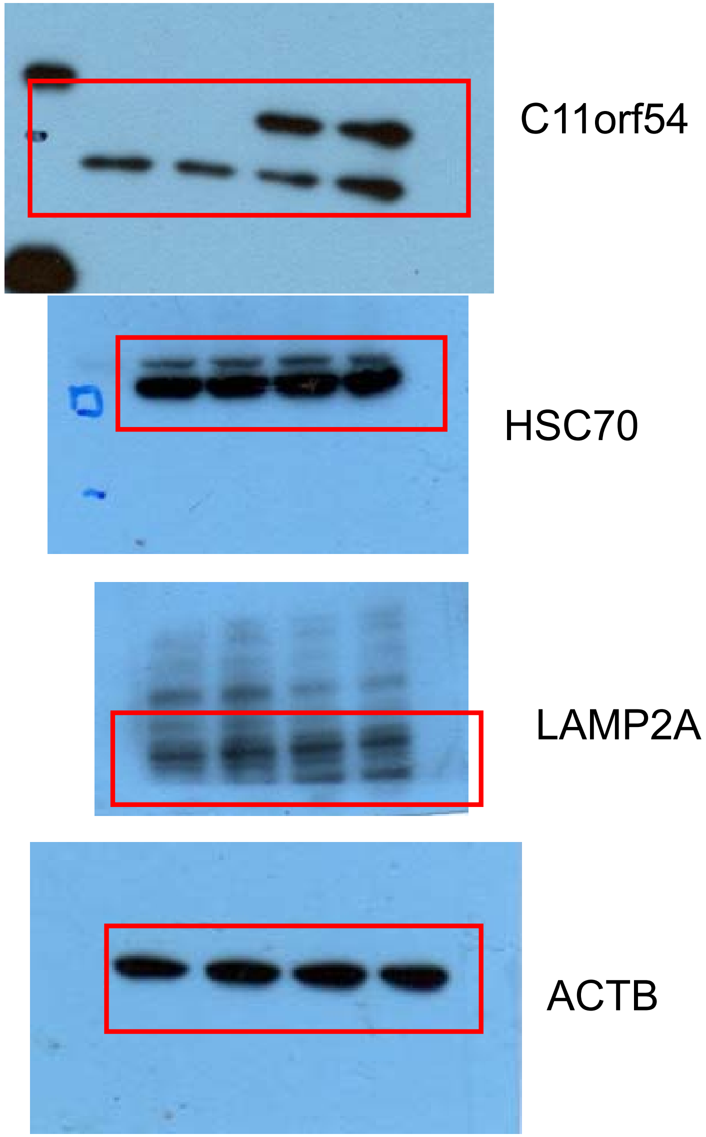

Supplementary Fig. 6d

Supplementary Table 1. Sequences of primers used in the present study

Gene name	Sequences, 5' to 3'
C11orf54-F	ATGGCTTGTGCTGAGTTTT
C11orf54-R	TTAATCTCGCCCAATTGAATGC
siLAMP2A	GCAGTGCAGATGACGACAAdTdT
Human-*ACTB*	GGCATCCTCACCCTGAAGTA
	GGGGTGTTGAAGGTCTCAAA
Human-*C11orf54*	TGTTATTCCATAGCACGCCCA
	ACAGCGCACCAAGTATGAACA
Human-*PKM2*	TGGAGAAACAGCCAAAGGGG
	GTGGAGTGACTTGAGGCTCG
Human-*LDHA*	CGATTCCGGATCTCATTGCCA
	TCAGCTGATCCTTTAGAGTTGCC
Human-*PGK1*	GCTGGACAAGCTGGACGTTA
	TGGGACAGCAGCCTTAATCC
Human-*FBP1*	ACCCCGCTAACAAGAAGAGC
	GTCTAACACGGCCTCCTTCC
Human-*PDHA1*	GGAGCTGCAGACTTACCGTT
	TGCTGTTCACCATCCTGTCC
Human-*PEPCK*	CTGGCCTGCGGCTTAACT
	GGCCCAGATCTCCACTAAGC
Human-*ENO1*	GGGAATCCCACTGTTGAGGT
	GTTCTAAGGCTTACCCTTCCCC

Human-*PDK3*	GAGCAATCCCAGCAGTGAAC
	TCACAGAGAGGACCACAGCAT
Human-*GLUT1*	CGGACCCTGCACCTCATAG
	TGGCCACGATGCTCAGATAG
Human-*GLUT2*	CCTGGAATTGACAGGACTCCC
	AGCACAGCAGTGATGACAGT
Human-*HIF1a*	GTGAAGACATCGCGGGGAC
	TGGCTGCATCTCGAGACTTTT
Human-*HIF1b*	CGACCAGGGCTGGATTTTGA
	CCTGGCAAACCGCTCCTTAT
Human-*TERT*	CCTGCGTTTGGTGGATGATTT
	TACTCAGGGACACCTCGGAC
Human-*TERF2*	TGGGTGGAAGAGGATGAACTG
	TTGACCCACTCGCTTTCTTCT
Human-*RRM1*	GGCCGCCAAGAACGAGT
	GCCTTGGATTACTTTCATGGTGAT
Human-*RRM2*	AGAAACGAGGACTGATGCCTG
	CGATGGTTTGTGTACCAGGTG
Human-*KU70*	GAAGCAAAAGGCCCAAGGTG
	AGCAGCTCCTGCTTCTTCAG
Human-*KU80*	CCCGGAAGAAGCGACCAAA
	TCATGGTAAAGCCCACGTCC
Human-*RAD52*	GTAGGGAGAGGCTCTGGACA
	GCAGGTGCTTAGGACCAAGT

Human-*ATM*	CGCAGCCTTGAGTCTGTGTA
	CTGGGAGTGTTTCTGCCACT
Human-*ATR*	GATTGCAGCAACTCCCTCCT
	TCCACAGCATGACCCATCAC
Human-*CHK1*	TCTTTCGAAGCCTCTCGCTC
	CACAGTCGGTGAAGCAGAGT
Human-*CHK2*	CTGAGGCTGCGGAGAGTG
	TTGGCATCGTGCTGGTAGAG
Human-*HSC70*	GCTTCCTTCGTTATTGGAGCC
	ACACAAGAGTAGGTGGTGCC
Human-*LAMP2A*	TGACGACAACTTCCTTGTGC
	AGCATGATGGTGCTTGAGAC

Supplementary Table 2. Antibodies used in the present study.

Antibody	Company (catalog number)	Application
Primary antibodies		
ACTB	Sigma (A8481)	WB, 1:10000
GAPDH	Cell Signaling Technology (2118)	WB, 1:10000
H3	Cell Signaling Technology (9715)	WB, 1:10000
C11orf54	Proteintech (23251-1-AP)	WB, 1:4000
		IF, 1:400
C11orf54	Invitrogen (PA5-78481)	WB, 1:4000
p-H2A.X (Ser139)	Cell Signaling Technology (9718)	WB, 1:1000
		IF, 1:200
PARP	Cell Signaling Technology (9532)	WB, 1:2000
Caspase3	Cell Signaling Technology (9662)	WB, 1:2000
Cleaved -Caspase3	Cell Signaling Technology (9664)	WB, 1:1000
Bax	Proteintech (50599-2-Ig)	WB, 1:2000
Bcl-2	Proteintech (12789-1-AP)	WB, 1:2000
ATM	Proteintech (27156-1-AP)	WB, 1:2000
p-ATM (Ser1981)	Cell Signaling Technology (5883)	WB, 1:2000
CHK1	Proteintech (25887-1-AP)	WB, 1:2000
p-CHK1 (Ser345)	Cell Signaling Technology (2348)	WB, 1:2000
CHK2	Proteintech (13954-1-AP)	WB, 1:2000
p-CHK2 (Thr68)	Cell Signaling Technology (2197)	WB, 1:2000

Ku70	Cell Signaling Technology (4588)	WB, 1:2000
Rad51	Proteintech (14961-1-AP)	WB, 1:4000
Rad51	Abcam (ab133534)	IF, 1:200
HIF1a	Cell Signaling Technology (36169)	WB, 1:2000
PKM2	Proteintech (15822-1-AP)	WB, 1:5000
HK2	Proteintech (66947-1-Ig)	WB, 1:5000
LDHA	Proteintech (19987-1-AP)	WB, 1:5000
PGAM1	Proteintech (16126-1-AP)	WB, 1:5000
PFKP	Proteintech (13389-1-AP)	WB, 1:5000
G6PD	Proteintech (25413-1-AP)	WB, 1:3000
p62	Sigma (P0067)	WB, 1:10000
LC3B	Sigma (L7543)	WB, 1:5000
HSC70	Proteintech (10654-1-AP)	WB, 1:5000
LAMP2A	Abcam (ab125068)	WB, 1:2000
Flag-tag	Sigma (F1804)	IP, 1:1000
RRM2	Proteintech (11661-1-AP)	WB, 1:5000
Secondary antibodies		
Alexa Fluor® 488-AffiniPure goat anti-rabbit IgG	Jackson ImmunoResearch	IF, 1:400
Alexa Fluor® 594-AffiniPure goat anti-rabbit IgG	Jackson ImmunoResearch	IF, 1:400
VeriBlot for IP Detection Reagent (HRP)	Abcam (ab131366)	IP, 1:1000

Supplementary Table 3. KFERQ-like motifs in C11orf54 by KFERQ finder V0.8

entry	status	protein_names	gene_names	length	motif	motif_start	motif_type
Q9H0W9	reviewed	Ester hydrolase C11orf54 (EC 3.1.-.-)	C11orf54 LP4947 PTD012	315	QKKVY	74	phos. act.
Q9H0W9	reviewed	Ester hydrolase C11orf54 (EC 3.1.-.-)	C11orf54 LP4947 PTD012	315	KEIKL	85	acetyl. act.
