# Supplementary material for: C11orf54 promotes DNA repair via blocking CMA-mediated degradation of HIF1A
Source: Commun Biol. 2023 Jun 5;6:606. doi: 10.1038/s42003-023-04957-1 (PMC10241914; doi:10.1038/s42003-023-04957-1)
Supplement: Supplementary file 5 — Reporting Summary [file 42003_2023_4957_MOESM5_ESM.pdf]

nature portfolio

Corresponding author(s): Qinghua Zhou

Last updated by author(s): May 6, 2023

Reporting Summary

Nature Portfolio wishes to improve the reproducibility of the work that we publish. This form provides structure for consistency and transparency in reporting. For further information on Nature Portfolio policies, see our Editorial Policies and the Editorial Policy Checklist.

Statistics

For all statistical analyses, confirm that the following items are present in the figure legend, table legend, main text, or Methods section.

n/a	Confirmed	
☐	☒	The exact sample size (*n*) for each experimental group/condition, given as a discrete number and unit of measurement
☐	☒	A statement on whether measurements were taken from distinct samples or whether the same sample was measured repeatedly
☐	☒	The statistical test(s) used AND whether they are one- or two-sided<br>*Only common tests should be described solely by name; describe more complex techniques in the Methods section.*
☒	☐	A description of all covariates tested
☐	☒	A description of any assumptions or corrections, such as tests of normality and adjustment for multiple comparisons
☐	☒	A full description of the statistical parameters including central tendency (e.g. means) or other basic estimates (e.g. regression coefficient) AND variation (e.g. standard deviation) or associated estimates of uncertainty (e.g. confidence intervals)
☐	☒	For null hypothesis testing, the test statistic (e.g. *F*, *t*, *r*) with confidence intervals, effect sizes, degrees of freedom and *P* value noted<br>*Give P values as exact values whenever suitable.*
☒	☐	For Bayesian analysis, information on the choice of priors and Markov chain Monte Carlo settings
☒	☐	For hierarchical and complex designs, identification of the appropriate level for tests and full reporting of outcomes
☒	☐	Estimates of effect sizes (e.g. Cohen's *d*, Pearson's *r*), indicating how they were calculated

Our web collection on statistics for biologists contains articles on many of the points above.

Software and code

Policy information about availability of computer code

Data collection	qPCR: Bio-Rad CFX, Microscopy: NIS-Elements(Nikon) or LAS AF Lite(Leica), Molecular cloning: SnapGene, FACS: FlowJo, Comet assay: CASP, Western blot protein band intensity: Image J
Data analysis	GraphPad Prism 9

For manuscripts utilizing custom algorithms or software that are central to the research but not yet described in published literature, software must be made available to editors and reviewers. We strongly encourage code deposition in a community repository (e.g. GitHub). See the Nature Portfolio guidelines for submitting code & software for further information.

Data

Policy information about availability of data

All manuscripts must include a data availability statement. This statement should provide the following information, where applicable:

- Accession codes, unique identifiers, or web links for publicly available datasets
- A description of any restrictions on data availability
- For clinical datasets or third party data, please ensure that the statement adheres to our policy

The source data are provided with this paper, all relevant data are available from the corresponding author upon any reasonable request.

Research involving human participants, their data, or biological material

Policy information about studies with human participants or human data. See also policy information about sex, gender (identity/presentation), and sexual orientation and race, ethnicity and racism.

Reporting on sex and gender	N/A
Reporting on race, ethnicity, or other socially relevant groupings	N/A
Population characteristics	N/A
Recruitment	N/A
Ethics oversight	N/A

Note that full information on the approval of the study protocol must also be provided in the manuscript.

Field-specific reporting

Please select the one below that is the best fit for your research. If you are not sure, read the appropriate sections before making your selection.

- [x] Life sciences
- [ ] Behavioural & social sciences
- [ ] Ecological, evolutionary & environmental sciences

For a reference copy of the document with all sections, see nature.com/documents/nr-reporting-summary-flat.pdf

Life sciences study design

All studies must disclose on these points even when the disclosure is negative.

Sample size	All data presented represent the mean ± s.d. of at least three biological replicates or three independent experiments. Difference between three or more means was assessed using One-way ANOVA.
Data exclusions	No data were excluded in our study.
Replication	Three or more independent experiments were performed or sufficient sample sizes were involved.
Randomization	The samples were randomly allocated into experimental group.
Blinding	For the GFP-LC3 puncta, the p-H2A.X foci analysis and comet assay experiments, the investigator were blinded to group allocation. For other experiments, such as WB, q-PCR .etc, the Investigators were not blinded during experiments,as the same investigator performed those experiments and analyzed the

Reporting for specific materials, systems and methods

We require information from authors about some types of materials, experimental systems and methods used in many studies. Here, indicate whether each material, system or method listed is relevant to your study. If you are not sure if a list item applies to your research, read the appropriate section before selecting a response.

Materials & experimental systems

n/a	Involved in the study	
[ ]	[x]	Antibodies
[ ]	[x]	Eukaryotic cell lines
[x]	[ ]	Palaeontology and archaeology
[x]	[ ]	Animals and other organisms
[x]	[ ]	Clinical data
[x]	[ ]	Dual use research of concern
[x]	[ ]	Plants

Methods

n/a	Involved in the study	
[x]	[ ]	ChIP-seq
[ ]	[x]	Flow cytometry
[x]	[ ]	MRI-based neuroimaging

Antibodies

Antibodies used	Primary antibodies<br>ACTB Sigma (A8481) WB, 1:10000<br>GAPDH Cell Signaling Technology (2118) WB, 1:10000

H3 Cell Signaling Technology (9715) WB, 1:10000
C11orf54 Proteintech (23251-1-AP) WB, 1:4000 IF, 1:400
C11orf54 Invitrogen (PA5-78481) WB, 1:4000
p-H2A.X (Ser139) Cell Signaling Technology (9718) WB, 1:1000 IF, 1:200
PARP Cell Signaling Technology (9532) WB, 1:2000
Caspase3 Cell Signaling Technology (9662) WB, 1:2000
Cleaved -Caspase3 Cell Signaling Technology (9664) WB, 1:1000
Bax Proteintech (50599-2-Ig) WB, 1:2000
Bcl-2 Proteintech (12789-1-AP) WB, 1:2000
ATM Proteintech (27156-1-AP) WB, 1:2000
p-ATM (Ser1981) Cell Signaling Technology (5883) WB, 1:2000
CHK1 Proteintech (25887-1-AP) WB, 1:2000
p-CHK1 (Ser345) Cell Signaling Technology (2348) WB, 1:2000
CHK2 Proteintech (13954-1-AP) WB, 1:2000
p-CHK2 (Thr68) Cell Signaling Technology (2197) WB, 1:2000
Ku70 Cell Signaling Technology (4588) WB, 1:2000
Rad51 Proteintech (14961-1-AP) WB, 1:4000
HIF1a Cell Signaling Technology (36169) WB, 1:2000
PKM2 Proteintech (15822-1-AP) WB, 1:5000
HK2 Proteintech (66947-1-Ig) WB, 1:5000
LDHA Proteintech (19987-1-AP) WB, 1:5000
PGAM1 Proteintech (16126-1-AP) WB, 1:5000
PFKP Proteintech (13389-1-AP) WB, 1:5000
G6PD Proteintech (25413-1-AP) WB, 1:3000
p62 Sigma (P0067) WB, 1:10000
LC3B Sigma (L7543) WB, 1:5000
HSC70 Proteintech (10654-1-AP) WB, 1:5000
LAMP2A Abcam (ab125068) WB, 1:2000
Flag-tag Sigma (F1804) IP, 1:1000
RRM2 Proteintech (11661-1-AP) WB, 1:5000
Secondary antibodies
Alexa Fluor® 488- AffiniPure goat anti-rabbit IgG Jackson ImmunoResearch IF, 1:400
Alexa Fluor® 594- AffiniPure goat anti-rabbit IgG Jackson ImmunoResearch IF, 1:400
VeriBlot for IP Detection Reagent (HRP) Abcam (ab131366) IP, 1:1000
ACTB Sigma (A8481) WB, 1:10000
GAPDH Cell Signaling Technology (2118) WB, 1:10000
H3 Cell Signaling Technology (9715) WB, 1:10000
C11orf54 Proteintech (23251-1-AP) WB, 1:4000 IF, 1:400
C11orf54 Invitrogen (PA5-78481) WB, 1:4000
p-H2A.X (Ser139) Cell Signaling Technology (9718) WB, 1:1000 IF, 1:200
PARP Cell Signaling Technology (9532) WB, 1:2000
Caspase3 Cell Signaling Technology (9662) WB, 1:2000
Cleaved -Caspase3 Cell Signaling Technology (9664) WB, 1:1000
Bax Proteintech (50599-2-Ig) WB, 1:2000
Bcl-2 Proteintech (12789-1-AP) WB, 1:2000
ATM Proteintech (27156-1-AP) WB, 1:2000
p-ATM (Ser1981) Cell Signaling Technology (5883) WB, 1:2000
CHK1 Proteintech (25887-1-AP) WB, 1:2000
p-CHK1 (Ser345) Cell Signaling Technology (2348) WB, 1:2000
CHK2 Proteintech (13954-1-AP) WB, 1:2000
p-CHK2 (Thr68) Cell Signaling Technology (2197) WB, 1:2000
Ku70 Cell Signaling Technology (4588) WB, 1:2000
Rad51 Proteintech (14961-1-AP) WB, 1:4000
HIF1a Cell Signaling Technology (36169) WB, 1:2000
PKM2 Proteintech (15822-1-AP) WB, 1:5000
HK2 Proteintech (66947-1-Ig) WB, 1:5000
LDHA Proteintech (19987-1-AP) WB, 1:5000
PGAM1 Proteintech (16126-1-AP) WB, 1:5000
PFKP Proteintech (13389-1-AP) WB, 1:5000
G6PD Proteintech (25413-1-AP) WB, 1:3000
p62 Sigma (P0067) WB, 1:10000
LC3B Sigma (L7543) WB, 1:5000
HSC70 Proteintech (10654-1-AP) WB, 1:5000
LAMP2A Abcam (ab125068) WB, 1:2000
Flag-tag Sigma (F1804) IP, 1:1000
RRM2 Proteintech (11661-1-AP) WB, 1:5000
Secondary antibodies
Alexa Fluor® 488- AffiniPure goat anti-rabbit IgG Jackson ImmunoResearch IF, 1:400

Alexa Fluor® 594- AffiniPure goat anti-rabbit IgG Jackson ImmunoResearch IF, 1:400
VeriBlot for IP Detection Reagent (HRP) Abcam (ab131366) IP, 1:1000
ACTB Sigma (A8481) WB, 1:10000
GAPDH Cell Signaling Technology (2118) WB, 1:10000
H3 Cell Signaling Technology (9715) WB, 1:10000
C11orf54 Proteintech (23251-1-AP) WB, 1:4000 IF, 1:400
C11orf54 Invitrogen (PA5-78481) WB, 1:4000
p-H2A.X (Ser139) Cell Signaling Technology (9718) WB, 1:1000 IF, 1:200
PARP Cell Signaling Technology (9532) WB, 1:2000
Caspase3 Cell Signaling Technology (9662) WB, 1:2000
Cleaved -Caspase3 Cell Signaling Technology (9664) WB, 1:1000
Bax Proteintech (50599-2-Ig) WB, 1:2000
Bcl-2 Proteintech (12789-1-AP) WB, 1:2000
ATM Proteintech (27156-1-AP) WB, 1:2000
p-ATM (Ser1981) Cell Signaling Technology (5883) WB, 1:2000
CHK1 Proteintech (25887-1-AP) WB, 1:2000
p-CHK1 (Ser345) Cell Signaling Technology (2348) WB, 1:2000
CHK2 Proteintech (13954-1-AP) WB, 1:2000
p-CHK2 (Thr68) Cell Signaling Technology (2197) WB, 1:2000
Ku70 Cell Signaling Technology (4588) WB, 1:2000
Rad51 Proteintech (14961-1-AP) WB, 1:4000
Rad51 Abcam (ab133534) IF, 1:200
HIF1a Cell Signaling Technology (36169) WB, 1:2000
PKM2 Proteintech (15822-1-AP) WB, 1:5000
HK2 Proteintech (66947-1-Ig) WB, 1:5000
LDHA Proteintech (19987-1-AP) WB, 1:5000
PGAM1 Proteintech (16126-1-AP) WB, 1:5000
PFKP Proteintech (13389-1-AP) WB, 1:5000
G6PD Proteintech (25413-1-AP) WB, 1:3000
p62 Sigma (P0067) WB, 1:10000
LC3B Sigma (L7543) WB, 1:5000
HSC70 Proteintech (10654-1-AP) WB, 1:5000
LAMP2A Abcam (ab125068) WB, 1:2000
Flag-tag Sigma (F1804) IP, 1:1000
RRM2 Proteintech (11661-1-AP) WB, 1:5000
Secondary antibodies
Alexa Fluor® 488- AffiniPure goat anti-rabbit IgG Jackson ImmunoResearch IF, 1:400
Alexa Fluor® 594- AffiniPure goat anti-rabbit IgG Jackson ImmunoResearch IF, 1:400
VeriBlot for IP Detection Reagent (HRP) Abcam (ab131366) IP, 1:1000

Validation

All commercial antibodies were validated by the manufacturer for the species and application used in this study. We used antibodies recommended by the manufacturer for the species and application.

Eukaryotic cell lines

Policy information about cell lines and Sex and Gender in Research

Cell line source(s)

PLC/PRF/5 and 293T cell lines were purchased from the Shanghai Cell Bank, Type Culture Collection Committee, Chinese Academy of Sciences.

Authentication

PLC/PRF/5 and 293T cell lines were purchased from the Shanghai Cell Bank, Type Culture Collection Committee, Chinese Academy of Sciences.

Mycoplasma contamination

All cell lines used in the study were regularly tested for mycoplasma contamination and were determined to be negative.

Commonly misidentified lines
(See ICLAC register)

No misidentified lines were used.

Flow Cytometry

Plots

Confirm that:

- [x] The axis labels state the marker and fluorochrome used (e.g. CD4-FITC).
- [x] The axis scales are clearly visible. Include numbers along axes only for bottom left plot of group (a 'group' is an analysis of identical markers).
- [x] All plots are contour plots with outliers or pseudocolor plots.
- [x] A numerical value for number of cells or percentage (with statistics) is provided.

Methodology

Sample preparation	Cell apoptosis was assessed by flow cytometry using the Annexin V-FITC Apoptosis Detection Kit (Beyotime) following the manufacturer's instructions. Briefly, cells after treatment were trypsinized, washed with PBS, resuspended in binding buffer, and incubated with staining solution(annexin V/PI =2:1)in the dark for 20 minutes at room temperature. Immediately after the annexin V/PI staining, fluorescence-activated cell sorting (FACS) analysis was performed using BD FACS VERSE.
Instrument	We used BD FACS VERSE to collect the data.
Software	The softwares FlowJo and GraphPad Prism 10 were used to collect and analyze data.
Cell population abundance	The abundance of relevant cell populations was showed in the associated figures and figure legends.
Gating strategy	A blank group without any treatment was as a negetive control, and two single staining groupswhich only stained by FITC or PI. Then the positive cell population was defined according to the negative cell population.

- [x] Tick this box to confirm that a figure exemplifying the gating strategy is provided in the Supplementary Information.
